# Identification and integrative analysis of ACLY and related gene panels associated with immune microenvironment reveal prognostic significance in hepatocellular carcinoma

**DOI:** 10.1186/s12935-021-02108-2

**Published:** 2021-08-03

**Authors:** Yunfeng Xu, Ze Zhang, Da Xu, Xin Yang, Lina Zhou, Ying Zhu

**Affiliations:** 1 Department of General Surgery, Huashan Hospital, Cancer Metastasis Institute, Fudan University, 12 Urumqi Road (M), Shanghai, 200040 China; 2 CAS Key Laboratory of Separation Science for Analytical Chemistry, Dalian Institute of Chemical Physics, Chinese Academy of Sciences, Dalian, 116023 China

**Keywords:** Hepatocellular carcinoma, ACLY, Fatty acid metabolism, Portal vein tumor thrombosis, Immune checkpoint, Immune infiltration, Prognosis

## Abstract

**Background:**

Cumulating evidence reveals the key role of aberrant lipogenesis and immunogenomic features in hepatocellular carcinoma (HCC). However, there are still obstacles in our understanding of the complicated interaction between metabolic reprogramming and tumor immune microenvironment.

**Methods:**

We compared metabolomic, transcriptomic and immunogenomic characteristics of portal vein tumor thrombosis (PVTT) and primary tumor to seek valuable markers. Human HCC samples with PVTT (n  =  28) was analyzed through ultra-high-performance liquid chromatography-mass spectrometry (UHPLC-MS). Transcript levels of mRNA in two cohorts from published database GEO (n  =  60) and TCGA (n  =  411) were downloaded to explore differentially expressed genes and functional enriched gene set. Evaluation of immune infiltration was estimated and validated from transcriptomic data in both cohorts through six immune deconvolution algorithms and in a high-resolution mode (CIBERSORTx). Survival analysis (Kaplan–Meier and multivariable Cox regression model) was performed to examine prognostic value of ACLY, related immune checkpoints and immune infiltration levels from TCGA cohort. LASSO regression was further conducted to determine a gene panel to further predict survival outcomes associated with ACLY.

**Results:**

We identified a novel signature, ATP citrate lyase, through transcriptomic and metabolomic approaches. We demonstrated that the metabolism adaptations in both fatty acid and cholesterol biosynthesis triggered by ACLY oncogenic activation. We illustrated the crucial function of ACLY in lipogenesis and its potential interaction with immune microenvironment. CD276, a promising target in immune checkpoint blockade, showed correlation to ACLY and differential expression in ACLY risk classification. Combination of ACLY, CD276 and immune infiltration level and a novel ACLY-associated panel from a predictive model retrieved from published database validated the prognostic value to risk stratification in patients with HCC.ACLY blockade to counteract metabolic activation and immunosuppressive status of the tumor microenvironment highlighted attractive prospect for translational application.

**Conclusions:**

We investigated ACLY and its indispensable role in metabolism, immune function and a prognostic gene panel in HCC. We anticipate that the multifaced role of ACLY may reveal the potential value for mechanistic research and combinational therapy, suggesting that the combination blockade of ACLY and immune checkpoints may work as a promising strategy.

**Supplementary Information:**

The online version contains supplementary material available at 10.1186/s12935-021-02108-2.

## Introduction

Hepatocellular carcinoma (HCC) is the sixth most common cancer and third leading cause of cancer-related death worldwide. Portal vein tumor thrombosis (PVTT) is a common phenomenon in HCC [1]. Approximately 10–40% of patients exhibit macroscopic PVTT when HCC is first diagnosed [2, 3]. HCC patients complicated by PVTT are characterized with an aggressive disease course indicating deteriorated condition, treatment difficulty, and poor prognosis [4, 5]. Moreover, PVTT is clinically associated with large tumor size, increased tumor number, higher tumor grade, worse Child–Pugh class, and higher serum alpha-fetoprotein (AFP) [6]. The Barcelona Clinic Liver Cancer (BCLC) staging system designates PVTT as advanced disease (BCLC class C) for which only systemic therapy is currently recommended [5, 7]. Conventionally, PVTT is regarded as metastatic nodes compared with the primary tumor.

The drastic reprogramming of their metabolic pathways is one of the most significant and most frequent features of cancer cells [8]. There is an increased demand for energy and macromolecules due to the high proliferation rate of cancer cells [9]. Increased glucose uptake and fermentation of glucose to lactate is the most common characteristic of this altered metabolism. This phenomenon is known as the Warburg Effect [10]. Accumulative evidence has shown that cancer cells undergoing metabolic pathway changes alongside metabolic pathways.

Comprehensibly, upregulation of de novo lipid synthesis is one of the most significant metabolic signs in cancer cells [8]. Lipid biosynthesis could be classified into two objectives: fatty acid biosynthesis and mevalonate pathway, and the latter contributes to cholesterol and isoprenoid synthesis [11, 12]. Enhanced lipid synthesis and/or uptake leads to rapid enlargement of tumor size and progression, while de novo synthesis of fatty acid is suppressed in most noncancerous cells. Activated fatty acid synthesis in malignancies helps to fuel membrane biogenesis through rapid proliferation of cancer cells and saturation of lipid on cell membrane, thereby affecting fundamental cellular processes including signal transduction, functional gene expression and therapy response [13]. Metabolic microenvironment in tumor cells is observed from substantial increase in both transcriptomic level and activation of various enzymes participating in fatty acid biosynthesis process [13].

ATP citrate lyase (ACLY) is a cytosolic homotetrameric enzyme that catalyzes the conversion of citric acid to acetyl-CoA and oxaloacetate of citrate and coenzyme A (CoA) with the corresponding hydrolysis of ATP to ADP and phosphate [14]. It is worth noting that ACLY acts as a strategic converge communicating both glycolytic metabolism and lipid biosynthesis. In several types of tumors, transcriptomic regulation of ACLY proves to be aberrantly activated [15–17], and its pharmacological or genetic inhibition significantly inhibited the proliferation and induced apoptosis of cancer cells [18, 19]. Growing evidence highlights ACLY’s central role in giving this enzyme a strong therapeutic potential as a key target for cancer treatment.

A novel way in anti-tumor therapy has been demonstrated by immunomodulatory agents blocking the programmed cell death protein 1(PD-1)/programmed death 1 ligand 1(PD-L1) pathway [20]. The selective expression of PD-L1 with dominant immune-suppressive activities in the tumor microenvironment (TME), promoting a more favorable tumor response-to-toxicity ratio, may be the underlying reason for the success of these agents [21]. To strengthen antitumor immunity, a deeper understanding of key mechanisms creating an immunosuppressive tumor microenvironment remains a major challenge [20, 22]. Growing evidence reveals that TME promotes improper metabolic reprogramming that dampens the role of T cells and thus affects the immune response to the antitumor and the progression of the tumor [23]. The energy supplement of cancer cells collectively determines the TME’s metabolic environment.

Here, we preliminary investigated characteristics in PVTT, primary HCC tumor and adjacent normal tissue in HCC patients through metabolome and transcriptome approaches. A key player, ACLY, supporting lipid de novo synthesis was identified in association with TME among differentially expressed genes. Meanwhile, immune infiltration estimation in tumor samples was exhibited in a landscape. Clinical statistics were also used to evaluate prognostic markers in HCC. The coparticipation of ACLY, immune checkpoints and TME finally reflect significant prognostic efficient and could be considered as a potential combination therapy target in the future.

## Materials and methods

### Overview of this study

**Fig. 1:**
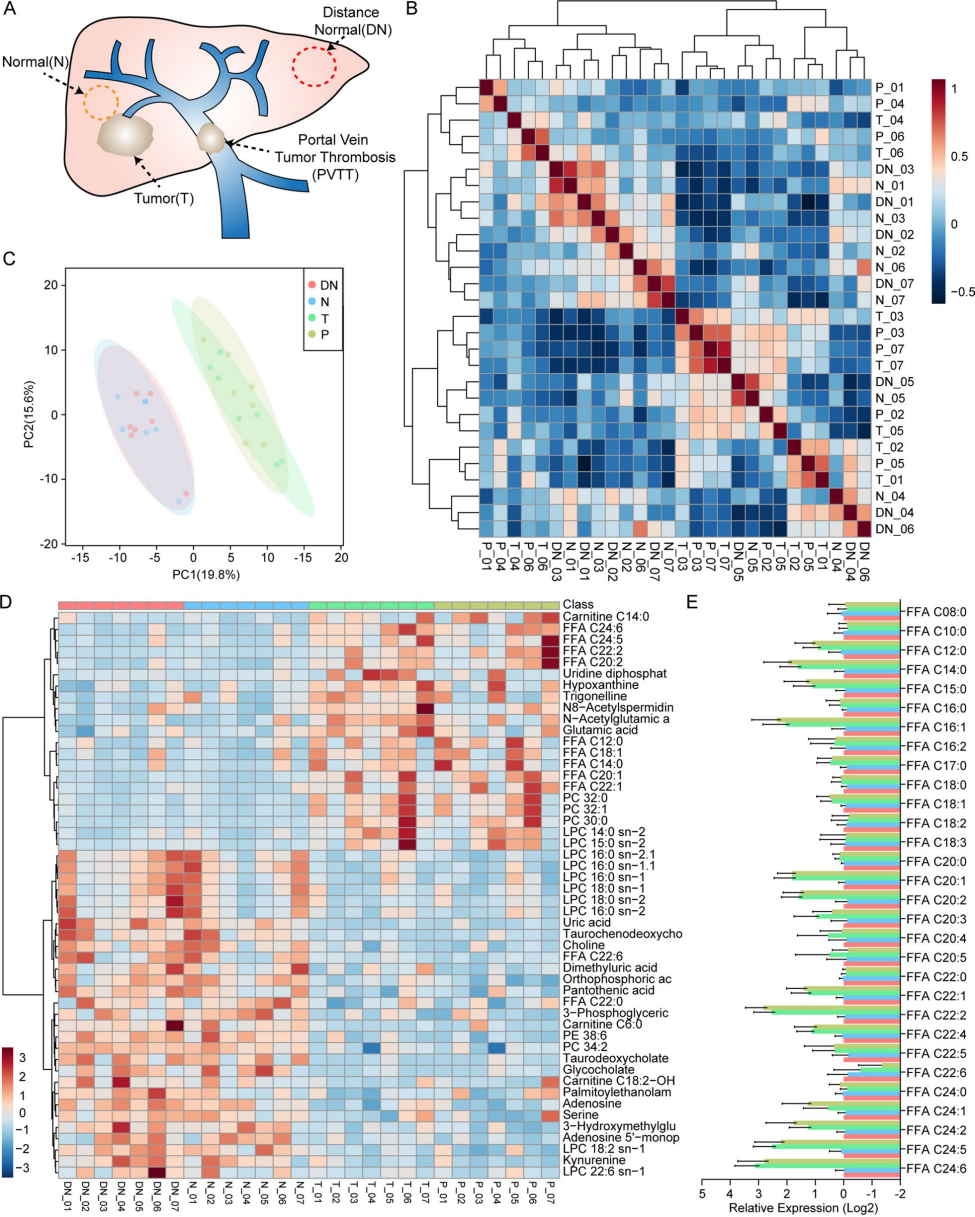
Metabolomic analysis of lipid metabolism in 7 HCC samples through UHPLC-MS. **A** Normal, distal noncancerous tissues, primary tumor and PVTT sample tissues were collected from 7 patients (labeled No.1–7) primarily diagnosed with hepatocellular carcinoma during in Huashan Hospital. **B** Heatmap showed the overall similarity of processed UHPLC-MS data. **C** PCA analysis indicated homogeneity of tumor and PVTT samples and homogeneity of normal and distal noncancerous samples in HCC. **D** Summary of all significantly differentially expressed metabolites in normal, distant normal, tumor and PVTT samples. **E** Overall FFA production rates was analyzed. *N* normal; *DN* distal normal; *T* tumor; *PVTT* portal vein tumor thrombosis; *PCA* principal component analysis; *UHPLC-MS* ultra-high-performance liquid chromatography-mass spectrometry; *FFA* free fatty acids; *PC* phosphatidylcholines; *LPC* lyso-PC; *PE* phosphatidylserines

In this study, metabolomic characteristics of HCC clinical samples were initially analyzed to illuminate the discrimination of lipid metabolites in PVTT, primary HCC tumor and adjacent normal tissue. We evaluated whole-transcriptomic data of 60 samples from 20 HCC patients (GSE77509) from Gene Expression Omnibus (GEO) dataset for validation (Fig. 1A). Patient (No.14) was excluded from the study, whose tumor and PVTT data were not distinguishable from normal tissue in pre-analysis. After identifying DEGs, enrichment analysis was performed to explore pathways and meaningful gene sets. Secondly, algorithms were used to estimate the tumor immune microenvironment of the patient samples, and high-resolution estimation of transcriptomic expression of immune cells classified by 10 subtypes in each sample were provided. Correlation analysis was performed to seek the significant relationship between TIICs, DEGs and representative markers of immune checkpoint. An integration of TIICs and DEGs was analyzed to further explore the potential mechanism between DEGs and TIICs, which could be used as a potential target for clinical medication. LASSO regression of univariate COX analysis was operated to seek for an ideal predictive model in HCC. To consolidate the clinical value of metabolomic and immunologic interaction and synergistic action, clinical data was applied to understand the prognostic value of metabolomic genes, tumor-infiltrating immune cells (TIICs) and differentially expressed genes (DEGs) separately or combinatorially.

### Clinical sample collection and tissue pretreatment for mass spectrometry

Our study was approved by the ethics committee of Huashan Hospital affiliate to Fudan University. Seven patients (labeled No.1–7) primarily diagnosed with HCC in Huashan Hospital were included in this study. Four types of tissue sample were obtained from each patient: primary HCC tumor, PVTT, adjacent normal tissue (ANT) and distal noncancerous tissue (DN). PVTTs were collected from portal vein. ANTs were collected from less than 2 cm into solid tumor border. DNs were collected from distal edge of the resected tissues, which were more than 2 cm from the border of HCC tumor. Consequently, a total of 28 tissue samples were prepared for mass spectrometry.

Pretreatment method was acquired from Zheng’s study [24]. The collected tissues were cut into pieces (100 mg) and mixed with 1 mL of 4:1 methanol/water and homogenized by high-speed blender. The sample were ultrasonicated and placed on ice for 20 min. Tissue samples (volume of each sample  ≤  200 μl) were prepared for defining MRM transitions through: centrifugation: 10 min at 15,000*g* and 4 ℃ after mix for 60 s; lyophilization: lypophilize the supernatant to a centrifuge tube and reconstruction: reconstitute the sample in 60 μl of 90% H_2_O/CH_3_OH, vortex for 60 s and centrifuge 10 min at 15,000*g* and 4 ℃.

### Metabolic profiling analysis based on ultra-high-performance liquid chromatography-mass spectrometry (UHPLC-MS)

Untargeted profiling was based on a Waters Acquity BEH C8 column to an AB Sciex Triple Q-TOF 5600  +  system by chromatographic separation and TOF 5600  +  mass spectrometer [24]. R packages XCMS [25] (http://www.bioconductor.org/packages/release/bioc/html/xcms.html) and CAMERA [26] (http://www.bioconductor.org/packages/release/bioc/html/CAMERA.html) were downloaded for peak detection and peak annotation. Five microliter of prepared reconstituted sample was then injected onto a UHPLC-HRMS with IDA mode for analyze. By applying MSConvert [27] (http://proteowizard.sourceforge.net/tools.shtml), the raw UHPLC-HRMS data format of some vendors was converted into XCMS-supported data types and mgf file type. Five microliter of IS reconstruction sample was injected onto the UHPLC-TQMS for retention-time calibration and CE voltage optimization. And the products were analyzed to evaluate the quantitative performance of pseudotargeted method in three prospects: linearity repeatability and stability. Unsupervised cluster analysis (Euclidean distance) and principal component analysis (PCA) were computed to discuss the divergence of samples.

### Evaluation of overall similarity of samples and data standardization

The chip datasets GSE77509 was downloaded from GEO database. The corresponding genes were transformed into Gene Symbol according to the annotation information on the platform. The first step is to confirm the heterogeneity of samples. Variance stabilizing transformation (VST), computed in R package DESeq2 [28] was performed to transform the homogeneity of variance. The clustering distance of overall similarity was also computed by Euclidean distance and PCA. IlluminaHiSeq data of 438 HCC samples in TCGA cohort was also downloaded and standardized for further analysis and comparison.

### Identification of candidate DEGs from GSE77509 data sets

DESeq2 was therefore used to perform differentially expressed gene analysis (DEA). The threshold was set by p value  <  0.0001, false discovery rate (FDR)  <  0.0001 and log_2_ fold change at least 1.5  ×  and all DEGs were illustrated in a volcano map. The candidate genes with significantly increased expression were marked with red line, and those with decreased expression were marked with blue line.

### Functional enrichment analysis and gene set enrichment analysis (GSEA)

For functional enrichment analysis, R package clusterProfiler [29] was applied to estimate enrichment in DEGs. Three biological characteristics: biological process (BP), cellular component (CC) and molecular functional (MF), were extracted from Gene Ontology (GO) enrichment analysis. The pathways discovered from DEGs were operated by matching the Kyoto Encyclopedia of Genome and Genome (KEGG). Top enrichment sets of each annotation were printed in barplot, dotplot and netplot by ggplot2 [30]. Then we applied GSEA software (v4.1.0) [31] to further assess enrichment of sets in GSE77509. GSEA input files were defined by expression data matrix, phenotype labels and annotation gene sets. GO and KEGG were also used as annotation gene sets. Sample classification [adjacent normal tissue (normal), primary tumor (tumor) and PVTT] were used as phenotype labels. And we analyzed the differential enrichment of Normal versus Tumor plus PVTT in GSE77509 cohort and tumor versus normal samples in TCGA HCC cohort by GSEA. The genes were rank-ordered by differential expression (signal2Noise) in the two phenotypes. And top 20 upregulated genes were showed in heatmap. Venn diagram [32] were used to compare the DEGs rank-ordered between GSEA ES score and DESeq2 FDR.

### Proteomic understanding of lipid metabolism associated DEGs and interaction networks

ACLY protein distribution in cells and expression in cancer were reviewed by using the Human Protein Atlas [33] (Images are available from v20.proteinatlas.org). Immunohistochemistry were adopted to show the distribution in cells and expression in cancer separately. STRING [34] (https://string-db.org/) was operated to further explore the co-expression network construction of lipid metabolism associated core gene set, as well as the network of 20 interactors in fatty acid biosynthesis process and 20 interactors in cholesterol biosynthesis process.

### Evaluation of immune infiltration in HCC

To evaluate immune infiltration and landscape of tumor immune microenvironment, whole transcriptomic expression data were uploaded in TIMER2.0 [35] (http://timer.comp-genomics.org/). Six immune deconvolution algorithms: TIMER (TMR) [36], CIBERSORT (CBS & CBS-ABS) [37], quanTIseq (QTS) [38], xCell (XCL) [39], MCP-counter (MCP) [40] and EPIC (EPC) [41] were used to estimate the infiltration condition of various kinds of immune cells. Landscape heatmaps in both GSE77509 and TCGA cohort were drawn to uncover the composition of TIICs of all the samples in six algorithms. And significant differential immune cells in MCP-counter and EPIC algorithms were showed respectively.

### Estimation of whole transcriptomic expression of immune cell in a high-resolution mode

Analytical tool CIBERSORTx [42] was applied to further provide the estimation of immune cell type specific transcriptomic profiles in a high-resolution mode. The types of immune cells in both GSE77509 and TCGA HCC cohort were merged into 10 major cell subsets. Batch correction was enabled to remove technical difference. The main result of CIBERSORTx High Resolution showed the cell-type specific expression of single genes at sample level. Genes were filtered by their geometric coefficient of variation (CV) calculated under the natural logarithm of subsampled regression coefficients. The geometric CVs were employed to determine the adaptive cell-type specific noise threshold. T-Distributed Stochastic Neighbor Embedding (t-SNE) was utilized for dimensionality reduction and visualization of transcriptomic data.

### Correlation analysis of metabolism associated DEGs, immune cell infiltration status, immune checkpoint inhibitors associated genes and prognostic gene panel

Correlation analysis were performed in both cohorts. Correlation was computed between lipid metabolism associated DEGs versus immune cell (CD8^+^ T cell, CD4^+^ T cell, Macrophage, Monocyte, Neutrophil and NK cell) infiltration status of 40 types of tumors. Correlation of lipid metabolism associated DEGs versus immune checkpoints was also assessed. Heatmaps were drawn to show the correlation result.

### Survival analysis and validation of prognostic significance of lipid metabolism associated DEGs and immune cell infiltration status

To validate the prognostic effectiveness of genes linked to lipid metabolism in HCC, clinicopathological characteristics of patients in TCGA cohort were enrolled for survival analysis due to the lack of clinical information in GSE cohort. According to the median of expression of lipid metabolism associated DEGs, the samples were divided into high expression group and low expression group. According to the median of TIICs estimation score or counts, the samples were divided into high immune infiltration group and low immune infiltration group. Through establishing multivariable Cox proportional hazard model (including patient age, tumor stage, patient gender, patient race and corrected tumor purity) and single gene expression, each classification was displayed in Kaplan–Meier curves. To further elucidate the relationship of gene expression and TIICs in HCC prognose, we then explore the combination of ACLY expression, immune checkpoint markers expression and TIICs status in Cox model. And Kaplan–Meier curves were drawn. The hazard ratio and p value for Cox model and the log-rank p value for KM curve were also provided.

### Determination of prognostic efficiency through establishment of a risk scoring model applying LASSO regression

The least absolute shrinkage and selection operator (LASSO) was fitted in R package glmnet (http://www.jstatsoft.org/v33/i01/) to select the genes serving as ideal prognostic factors in HCC. Univariate cox analysis was conducted to evaluate candidate gene. Candidates who meet the PH assumptions (ph  >  0.05) and significance analysis (z-score  <  0.05; p  <  0.05) of the cox equal proportional hazard model were selected for multivariate COX regression model. In the secondary modeling, variance inflation factor (VIF) and correlation coefficient of each factor in the LASSO model were calculated for collinearity analysis, and the COX regression forest map was drawn. Risk scores were accumulated by using coefficients scored from the model (Linear transformation calculation referencing the corresponding β coefficient, and the formula was: risk score  =  gene1*β1  +  gene2*β2…). Concordance index and time-dependent ROC curves were performed to appraise the predictive accuracy of the gene panel. Kaplan–Meier curves and log-rank test were executed to illustrate the prognostic efficiency.

### Statistical analysis

Experiments were repeated no less than three times. Statistical analyses for UHPLC-MS, expression level and immune infiltration level were performed using GraphPad Prism 8.0 software (GraphPad Software Inc., CA, USA). Transcriptomic level and TIIC infiltration level between or among groups ware computed by Student’s t test, nonparametric Mann–Whitney U-test and analysis of variance. Spearman correlation coefficient was conducted to evaluate association among gene expression and/or infiltration level. Data were presented as mean  ±  standard deviation. p values  <  0.05 were considered to be significant.

## Results

### Metabolomic overview of lipid profile in HCC clinical samples

To begin with, UHPLC-MS was performed to understand the metabolic condition in PVTT samples compared with normal tissues and HCC tissues in clinical samples. Similarity analysis results uncovered that PVTT and HCT were highly clustered (Fig. 1B). By using PCA analysis, we found that there was no significant difference between PVTT and tumor tissue in metabolomics level (Fig. 1C). We then characterized metabolism products expression in Fig. 1D. Detailed production rates of free fatty acids (FFA) with different carbon chain lengths were concluded in Fig. 1E, illustrating that metabolists in fatty acid biosynthesis, especially free fatty acids, increased in tumor and PVTT tissues, implying an activation of de novo synthesis in fatty acids.

### Overall similarity analysis and qualification of samples in GSE77509

**Fig. 2:**
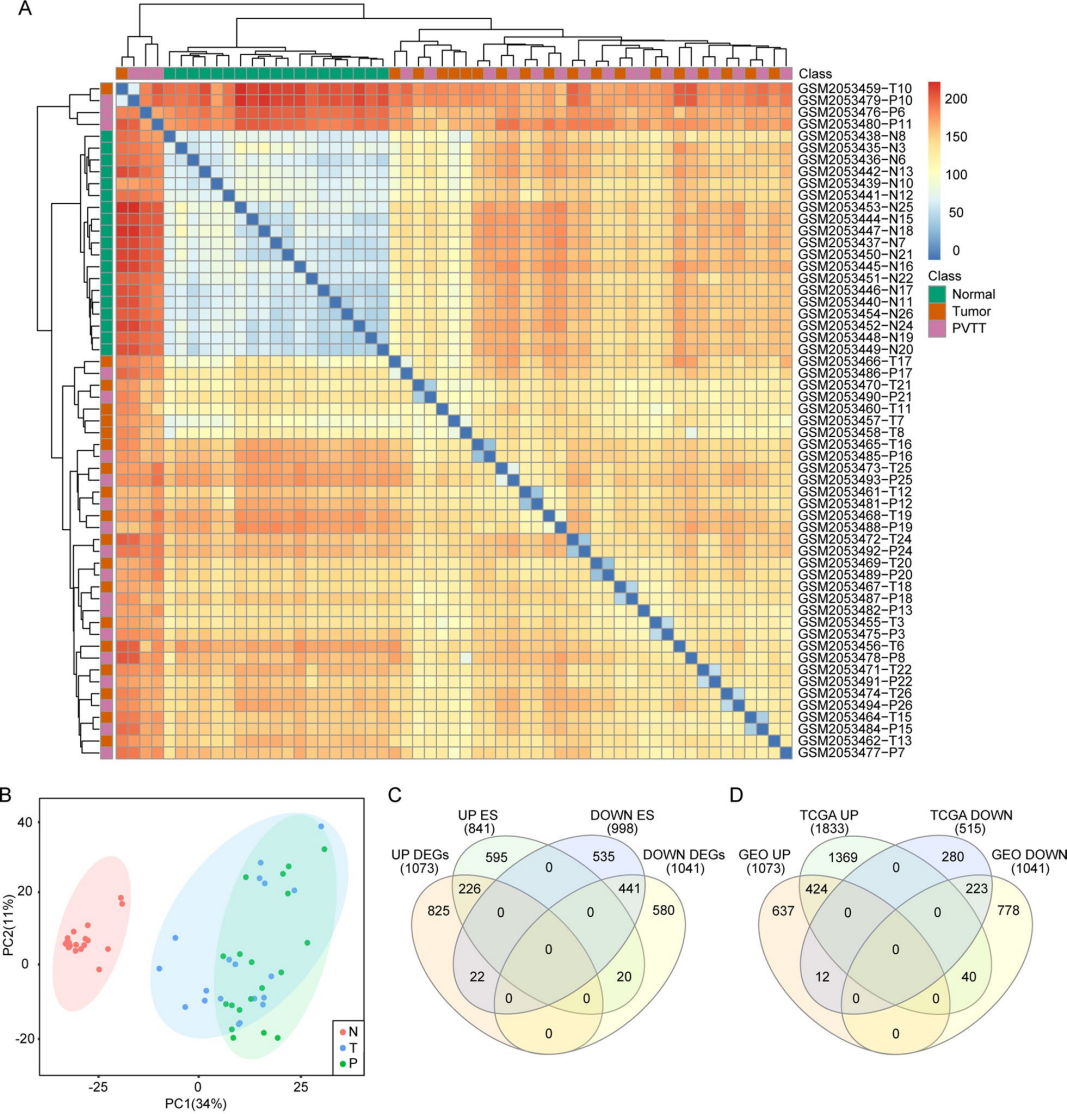
Transcriptomic characteristics of samples and summary of DEGs in GSE77509. **A** Heatmap showed the clustering results of normal, tumor and PVTT samples. **B** PCA analysis indicated homogeneity of tumor and PVTT samples. **C** Identification of upregulated and downregulated DEGs through fold change (| log_2_(fold change) |> 1.5) and FDR p value (p  <  0.00001) by DESeq2 and ES score (| ES |> 1) computed by GSEA in two cohorts

Expression data in published database was cited to compare and validate the difference between tumor and PVTT. Except for one tumor tissue (GSM2053457-T7), all adjacent normal tissues were clustered together by unsupervised hierarchical clustering (Fig. 2A). Dimensionality reduction analysis showed that there was no significant difference between primary tumor tissue and PVTT samples, and all normal tissue were distinguished from tumor tissue (Fig. 2B), suggesting that the chip data were qualified for following statistical process. Though PVTT was considered as a metastasis sign, given the overall metabolomic and transcriptomic performance, we didn’t find any meaningful characteristics in PVTT.

### Identification of candidate DEGs and gene enrichment score analysis

**Fig. 3:**
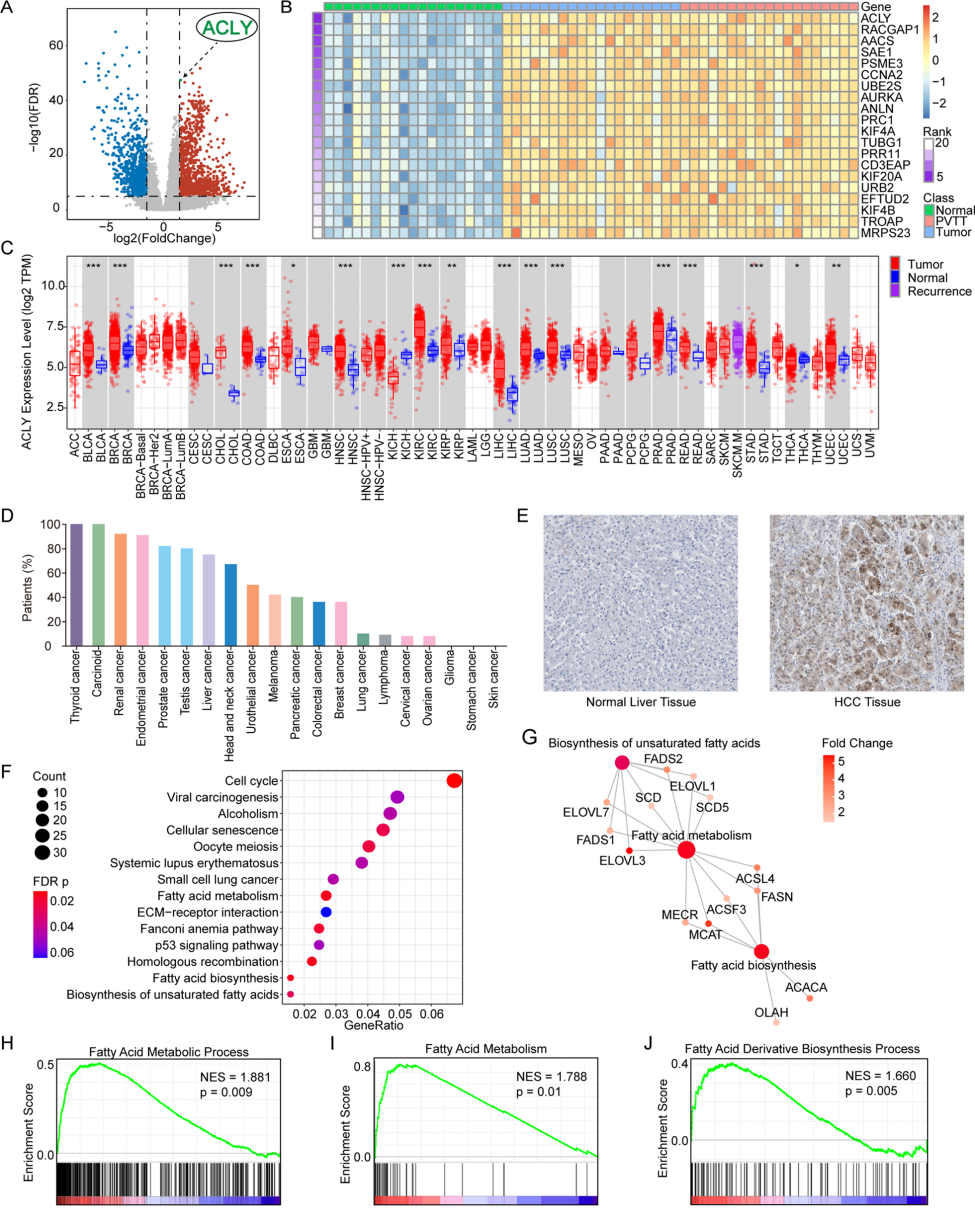
ACLY was a top differently expressed gene in HCC and other solid tumors. **A** Volcano plots showing DEGs in GSE77509 cohort. ACLY was an upregulated signature. **B** Top 20 markers identified by GSEA enrichment analysis: ACLY was at the top of the list. **C** An overview of ACLY expression of 40 solid tumors in TCGA dataset. **D** Proteomic ACLY expression rate of 20 solid tumors in HPA database. **E** Immunohistochemistry stained the expression of ACLY in primary HCC and adjacent normal tissue in HPA database. **F** Function enrich analysis indicated the top enriched pathways by KEGG annotation: fatty acid metabolism, fatty acid biosynthesis and biosynthesis of unsaturated fatty acids were positively enriched. **G** Interaction of fatty acid metabolism associated genes. **H** GSEA enrichment plots revealed activation in fatty acid metabolic process and fatty acid derivative biosynthesis process by GO annotation, along with fatty acid metabolism by KEGG annotation

1073 upregulated DEGs and 1041 downregulated DEGs were screened in tumor tissue and PVTT together compared with normal tissue in GSE77509 (Fig. 3A) and TCGA cohort (Additional file 1) by using DESeq2 package. According to the FDR p value, the top 5 upregulated DEGs were UBE2V2, RAD17, ACLY (labeled in Fig. 3A), UCN and KPNA2. The top 5 downregulated DEGs were PTHLH, ADAMTS1, CPEB3, STAC3 and CETP. GSEA analysis was employed so that a gene list rank-ordered with enrichment score by signal2Noise was given to further validate the DEGs. 841 positively enriched genes and 998 negatively enriched genes were identified, among which ACLY, RACGAP1, AACS and SAE1 were of the enrichment score (ES)  >  1.80 and OIT3, DNASE1L3, ECM1, BMPER and NDST3 were with the enrichment score (ES)  <  − 2.80. We summarily identified 226 DEGs in both upregulated groups and 441 DEGs in two downregulated groups. All DEGs were summarized in Venn diagram (Fig. 2A, B). Top 20 genes with the highest enrichment score were displayed in heatmap (Fig. 3B).

### Transcriptome and proteome expression of ACLY in HCC

Among the DEGs enriched at the top of the list, core gene ACLY associated with metabolism should be taken into serious account for further analysis. TIMER2.0 database was referred to show the transcriptome expression of ACLY across all TCGA tumors. Statistical significance was computed under Wilcoxon test, and ACLY expression was significantly higher (p  <  0.001) in various types of tumor including HCC (Fig. 3C). To understand the proteome expression of ACLY, we performed data from HPA database and showed distribution of ACLY protein expression in normal tissue and different types of malignancies. ACLY was often with low level of protein expression in normal liver tissue among all organ tissues. However, approximately 80% of the patients in HCC indicated an increase in ACLY protein expression (Fig. 3D). Immunohistochemistry also showed increased ACLY expression in tumor tissue compared with normal tissue (Fig. 3E).

### Upregulation of lipid metabolism associated with ACLY

The next part was to explain whether ACLY was involved or interacted in fatty acid metabolism and/or cholesterol metabolism. To gain insight into the functional role of upregulated DEGs in tumor and PVTT tissue in GSE77509, we exploited GO and KEGG annotation to determine the potential pathway and enrichment gene set. Gene sets with the most enriched gene numbers and least p values were listed in Additional file 1. Related genes in enriched gene sets were associated with GO Molecular Function: ATPase activity, DNA replication origin binding, cyclin-dependent protein serine/threonine kinase regulator activity, fatty acid synthase activity, glucose binding, biosynthesis of unsaturated fatty acids, etc. and cell cycle, fatty acid biosynthesis, fatty acid metabolism, homologous recombination, p53 signaling pathway in KEGG annotation (Fig. 3F; Additional files 1; 2: Figure S2A, B). Moreover, GSEA analysis consolidated that there was an obvious increase in fatty acid de novo biosynthesis and metabolism process (Fig. 3H–J).

**Fig. 4:**
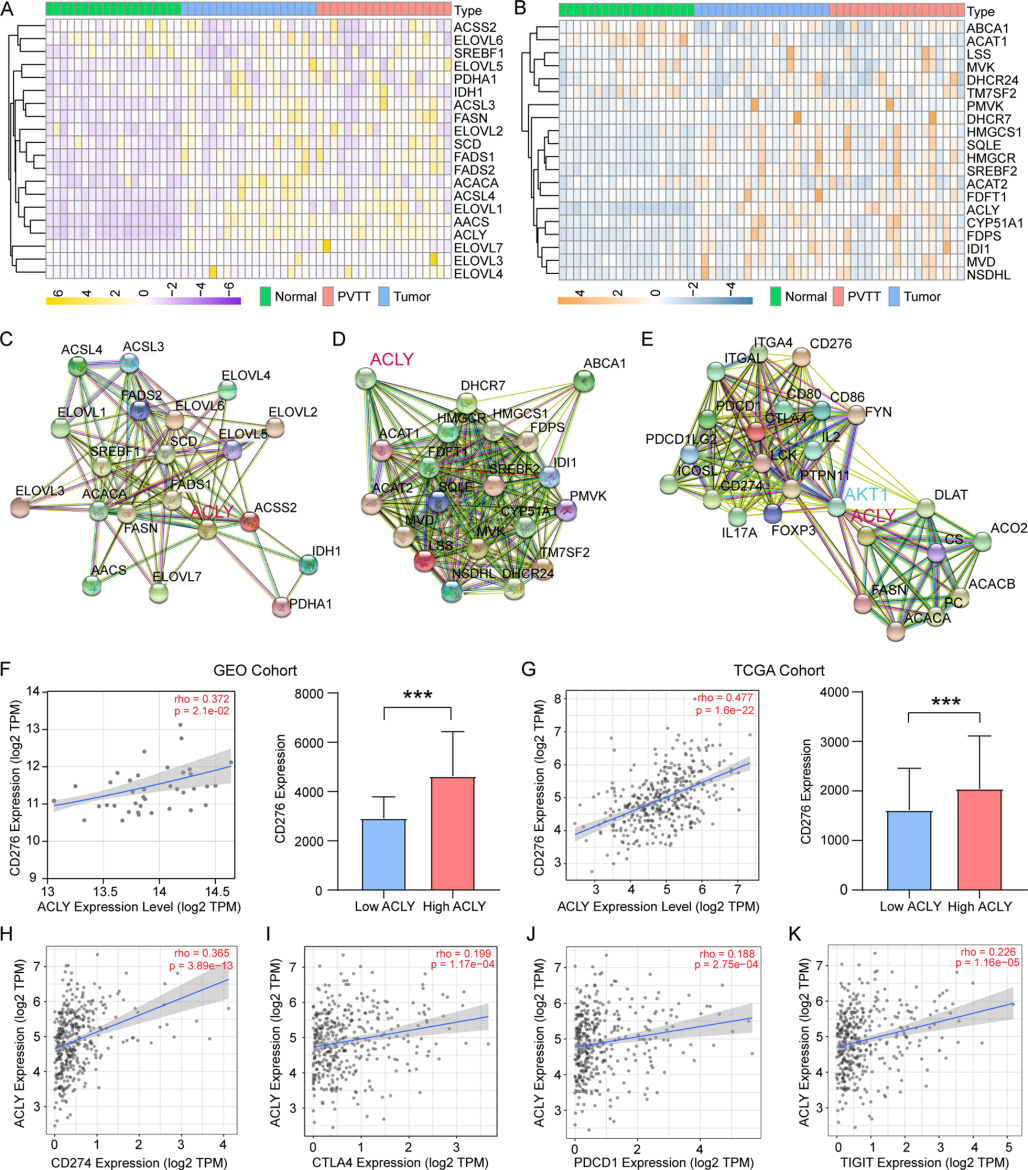
ACLY acted as an upstream regulator had close association with fatty acid and cholesterol biosynthesis and correlated with immune checkpoints in HCC. **A** Expression of fatty acid biosynthesis pathway related genes. **B** Expression of cholesterol biosynthesis pathway related genes. **C** Proteomic interactions between ACLY and core participants in fatty acid biosynthesis. **D** Proteomic interactions between ACLY and core participants in cholesterol biosynthesis. **E** Communication between lipid metabolism and immune checkpoints induced by ACLY and AKT1. **F**, **G** Correlation of ACLY and CD276 transcriptomic level and differentiated expression of CD276 in high ACLY expression and low ACLY expression group in both GSE77509 and TCGA cohort. **H**–**K** Other clinically approved and potential immune checkpoints correlated with ACLY in TCGA cohort

According to GO and KEGG enrichment analysis results, core genes in lipid metabolism, such as FASN, ACACA, ELOVLs, FADS2 and SCD, especially fatty acid metabolism: fatty acid biosynthesis, elongation and unsaturation, were involved in the enrichment analysis. We then selected core genes in fatty acid biosynthesis process and cholesterol biosynthesis process separately through enrichment analysis results, DEGs, reviews and former studies [43]. The overall expression of targeted genes in GEO77509 cohort and TCGA HCC cohort (50 tumor tissue samples and 50 homologous adjacent normal tissue samples) in relation to fatty acid biosynthesis process and cholesterol biosynthesis process in HCC were drawn in heatmaps (Fig. 4A, B; Additional file 3: Figure S3A, B). As ACLY was not included in the annotation of fatty acid metabolism associated pathways and gene sets in GO and KEGG, we analyzed the correlation between ACLY and 20 core genes in lipid metabolism process through TIMER in TCGA tumor cohort. ACLY had positive correlation with almost all core genes in fatty acid biosynthesis process (Spearman’s rho  >  0.2; p  <  0.05, except ACSS2; Additional file 4: Figure S4A) and cholesterol biosynthesis process (Spearman’s rho  >  0.2; p  <  0.05, except TM7SF2; Additional file 4: Figure S4B). ACAT1 and TM7SF2 were negatively correlated with ACLY (Spearman’s rho  <  − 0.1; p  <  0.05; Additional file 4: Figure S4B). Correlation analysis of 20 genes in fatty acid biosynthesis process and cholesterol biosynthesis process in other tumors in TCGA cohort were also demonstrated in Additional file 4: Figure S4A, B. Functional protein association networks validated the interaction between ACLY and two downstream metabolic pathways (Fig. 4C, D), which confirmed that ACLY was a core upstream marker in fatty acid biosynthesis. Figure 4E illustrated the probable proteomic communication between ACLY and immune checkpoints inducing by AKT pathway.

### Correlation of ACLY and immune checkpoints

Spearman correlation analysis was computed to measure the relevance of ACLY expression and immune checkpoints in both cohorts. As is depicted in Fig. 4F–M, clinically approved and potential immune checkpoint gene markers were also interrelated with ACLY in transcriptome level. ACLY has remarkable correlation with CD276 (Spearman’s rho  =  0.477; p  <  0.001), PDCD1 (Spearman’s rho  =  0.188, p  <  0.001), CD274 (encoding PD-L1) (Spearman’s rho  =  0.365, p  <  0.001) and CTLA4 (Spearman’s rho  =  0.199, p  <  0.001) in TCGA database and CD276 (Spearman’s rho  =  0.372, p  =  0.021) in GSE77509 cohort. Overall correlation analysis with immune checkpoints was illustrated in Additional file 5: Figure S5. Patients were divided into low and high expression group by median expression of ACLY. It is noteworthy that CD276 expression was also higher in high ACLY group (Fig. 4G, I), illustrating the worthy association of immune checkpoints and ACLY in HCC.

### Landscape of immune infiltration in HCC

**Fig. 5:**
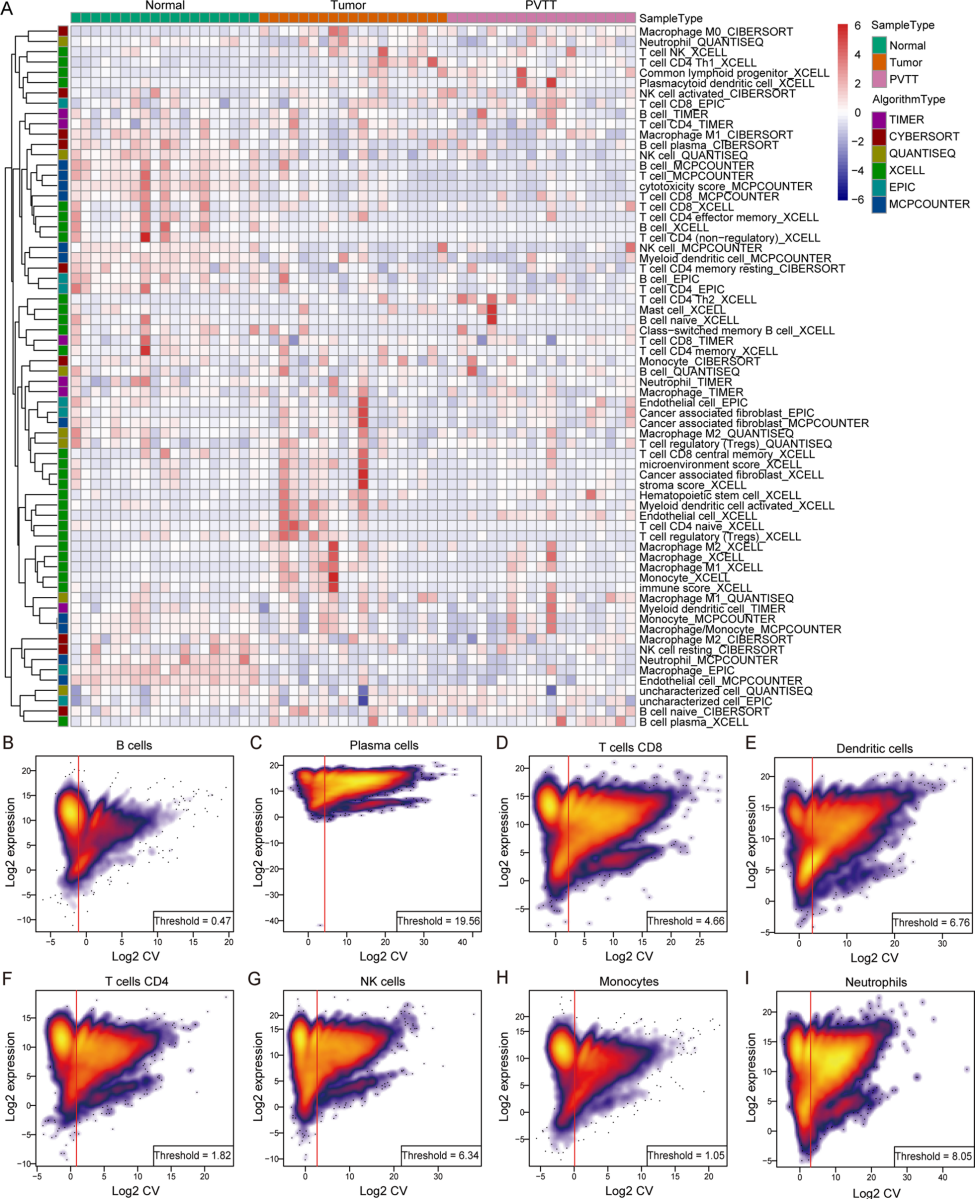
Immune landscape in HCC and expression distribution in 10 types of TIIC **A** Heatmap showed the immune infiltration status between normal, tumor and PVTT samples. The estimation of TIICs was computed and estimated by six kinds of algorithms based on the transcriptomic RNA-seq data in GSE 77509. **B**–**I** Normalized gene expression were plotted and geometric CV were computed to determine the adaptive cell-type specific noise threshold. **J** Estimated expression of ACLY in different types of immune cells in published datasets. **K** Estimated expression of CD276 in different types of immune cells in published datasets

To clarify the rule of TIIC infiltration and relationship with ACLY, we applied the six algorithms mentioned above to evaluate immune cell infiltration in GSE77509 and TCGA cohort. Landscape of detailed information of TIIC condition in GEO cohort was painted in heatmap (Fig. 5A and TCGA cohort Additional file 6: Figure S6). Unsupervised hierarchical clustering result categorized samples into three categories: immune inflamed, immune excluded and immune desert which has been defined in former studies [44–46] (Additional file 7; Additional file 8). MCP and EPC are the two algorithms recommended in the comparison of transcriptome-based cell-type quantification methods for immuno-oncology, which were qualified for most types of immune cells in evaluation. Therefore, the results revealed that the infiltration level of B cell (MCP-p  <  0.001, EPC-p  <  0.05; Additional file 9: Figure S9A, B), macrophage (MCP-p  <  0.001, EPC-p  <  0.001; Additional file 9: Figure S9C, D) and were increased in adjacent normal tissue rather than primary tumor tissue and PVTT in both algorithms. NK cell (p  <  0.001), CD8  +  T cell (p  <  0.001) and myeloid dendritic (p  <  0.001) cell (Additional file 9: Figure S9E–G) were higher in normal tissue estimated in MCP while CD4  +  T cell (p  <  0.01; Additional file 9: Figure S9H) was lower expressed in tumor tissue through calculation in EPC. In TCGA cohort, ACLY expression were positively associated with CD4^+^ T cell (EPC, TMR, XCL and CBS) (Additional file 10: Figure S10A), monocyte (MCP and QTS) (Additional file 10: Figure S10B), mDC (TMR, MCP and CBS) (Additional file 10: Figure S10C), Treg (CBS, QTS and XCL) (Additional file 10: Figure S10D) and neutrophil (TMR, CBS, MCP and QTS) (Additional file 10: Figure S10E) with p value  <  0.05. ACLY expression were negatively related to CD8^+^ T cell (CBS and XCL) (Additional file 10: Figure S10F) and NK cell (EPC and CBS) (Additional file 12: Figure S12G) with p value  <  0.05. However, the relationship of macrophage and TIICs varied in six algorithms among which EPC, XCL had negative correlation and TMR, QTS, XCL and MCP had positive correlation (Additional file 10: Figure S10H). In GSE77509 cohort, ACLY expression was negatively correlated with NK cell (MCP-p  =  0.003, CBS-p  =  0.040) and CD8^+^ T cell (XCL p  =  0.026).

### High-resolution analysis of transcriptomic expression of specific TIICs

**Fig. 6:**
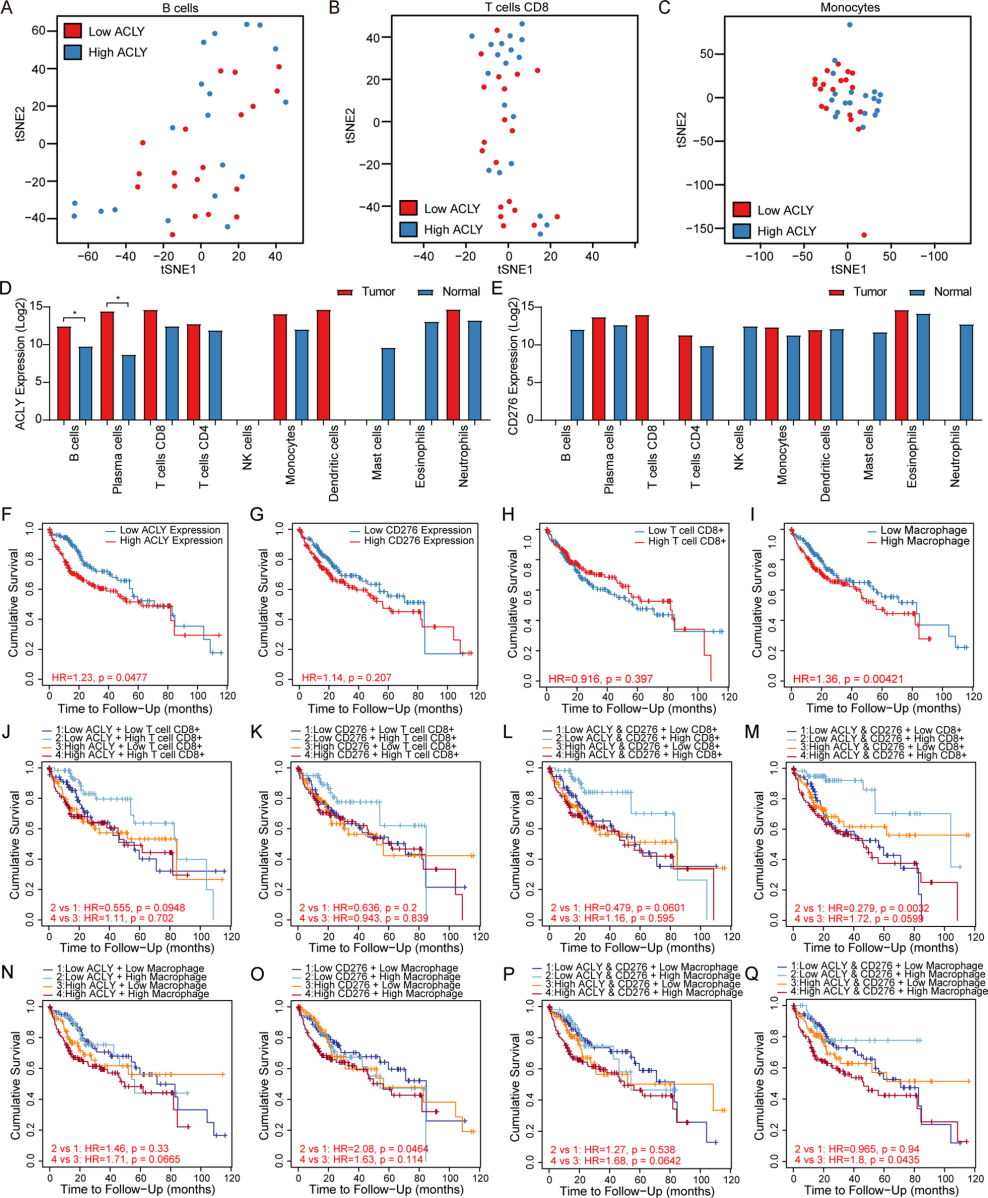
High-resolution estimation of ACLY in TIICs. ACLY combinated with immune checkpoints and TIICs elucidated strong prognostic value. **A**–**C** Distribution of samples through t-SNE analysis classified by ACLY expression. **D**, **E** Estimated transcriptomic level of ACLY and CD276 in cell-type specific view by CIBERSORTx. The NA values are genes that are either not expressed or have inadequate statistical power to be imputed. **F**, **G** Kaplan–Meier curves showing outcomes of overall survival between patients with high and low expression of ACLY and CD276. **H**, **I** Survival outcomes between patients having high and low levels of CD8^+^ T cells and macrophages. **J**–**M** Kaplan–Meier plot showed significant differences combining ACLY and CD276 expression with estimated infiltration levels of CD8^+^ T cells compared with single gene expression. **N**–**Q** No significant differences was observed combining ACLY and CD276 expression with estimated infiltration levels of macrophage

Further approaches were utilized to estimate the transcriptomic expression of 10 types of immune cells using CIBERSORTx in GEO cohort (Fig. 5B–I) and TCGA cohort (Additional file 11: Figure S11). ACLY was estimated to be a potential marker highlighting CD8^+^ T cell, CD4^+^ T cell, B cell and monocyte in two cohorts. The results of t-SNE analysis indicated that ACLY expression could not be applied to classify TICCs (Fig. 6A–C; Additional file 12: Figure S12). Top 1000 upregulated DEGs in both cohorts were drawn in heatmaps in Additional file 13: Figure S13. Estimated ACLY and CD276 expression were available in Fig. 5L, M. Higher expression level of ACLY was observed in B cell (p  =  0.049) and plasma cell (p  =  0.049). No statistic difference was observed in CD276 expression. The missing blank indicated that the expression level in specific immune cells lack reliability.

### Prognostic application of core genes in fatty acid biosynthesis

**Fig. 7:**
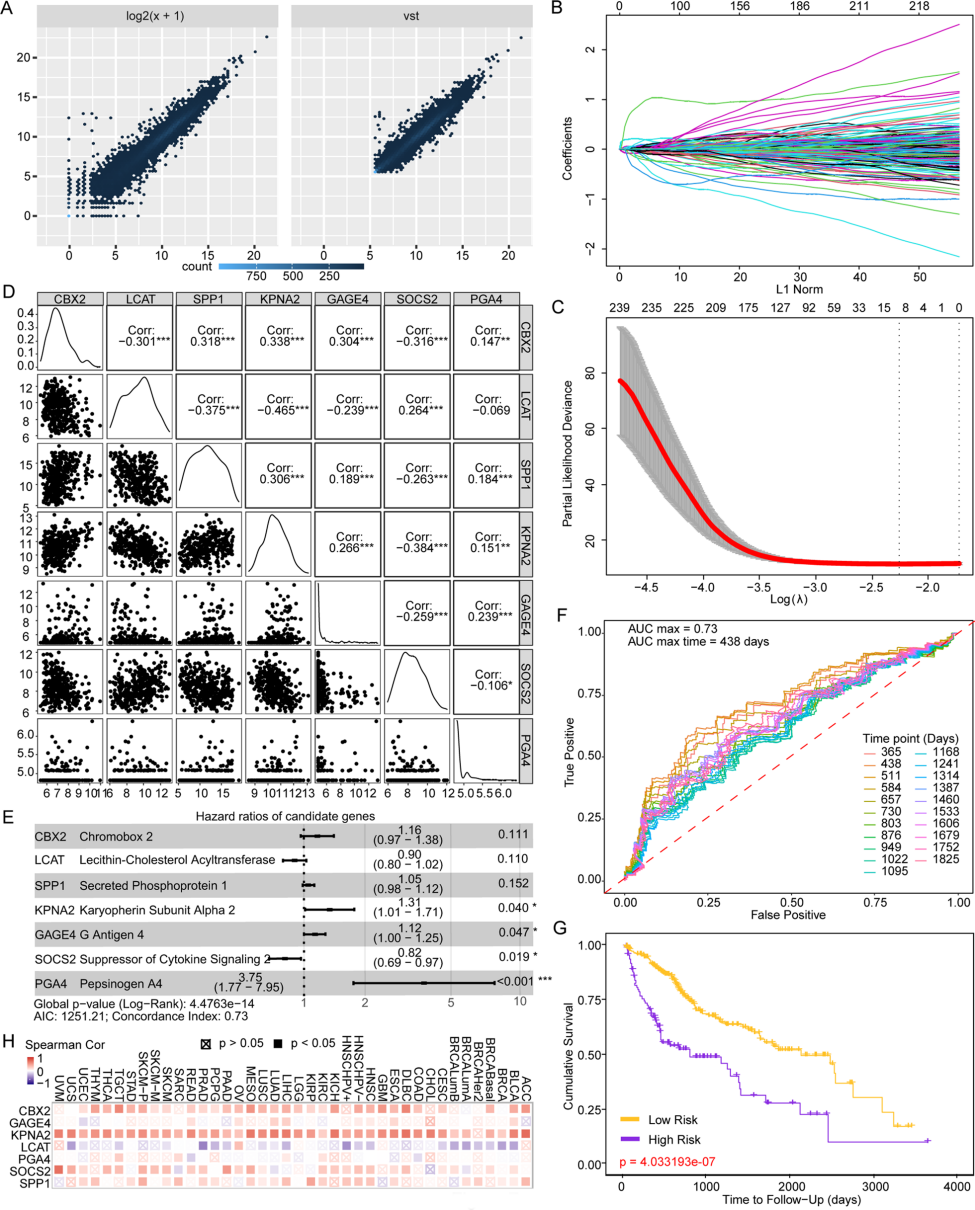
A novel gene signature in predicting survival outcomes and its relationship with ACLY. **A** VST transformation of DEGs for further model establishment. **B** LASSO coefficient of 4559 candidates. **C** Partial likelihood deviance was computed for LASSO regression. **D** Correlation matrix revealed no multicollinearity among 7 candidate genes. **E** Forest plot demonstrated HR and p value of multivariate Cox model enrolled genes. **F** Time-dependent ROC curves of different time length for optimal observation time window. **G** Kaplan–Meier survival analysis between patients with high-risk scores and low risk scores. **H** Association of ACLY with the predictive signature in 40 kinds of solid tumors

We next implemented survival analysis to discover powerful predictors in HCC. Multivariable Cox proportional hazard model was established. Patients with high expression of ACLY experienced worsen survival outcomes compared with low expression group (Cox HR  =  1.397, 95% CI 1.096–1.780; p  =  0.007, Kaplan–Meier curves: HR  =  1.23, p  =  0.048; Fig. 7I), while CD276 did not show any predictive value (Fig. 7J). In fatty acid biosynthesis process, AACS (Cox HR  =  1.380, 95% CI 1.110–1.716; p  =  0.004; KM curve: HR  =  1.32; p  =  0.009), ACACA (Cox HR  =  1.455, 95% CI 1.120–1.890; p  =  0.005, KM curve: HR  =  1.15; p  =  0.194), ACSL3 (Cox HR  =  1.316, 95% CI 1.038–1.668; p  =  0.023, KM curve: HR  =  1.25; p  =  0.032), ELOVL1 (Cox HR  =  1.699; 95% CI 1.268–2.276; p  <  0.001; KM curve: HR  =  1.35; p  =  0.005), ELOVL3 (Cox HR  =  1.502; 95% CI 1.125–0.005; p  =  0.006, KM curve: HR  =  1.23; p  =  0.047) and ELOVL4 (Cox HR  =  1.623, 95% CI 1.152–2.286; p  =  0.006, KM curve: HR  =  1.35; p  =  0.006) were also proved to be significant markers in predicting clinical survival outcomes (Additional file 14: Figure S14). High expression of mentioned genes was related to poor prognosis. In cholesterol biosynthesis process, only high expression of ACAT1 was correlated with better prognosis (Cox HR  =  0.778, 95% CI 0.648–0.935; p  =  0.007, Kaplan–Meier curves: HR  =  0.817; p  =  0.053; Additional file 14: Figure S14).

### Predictive performance of ACLY expression combined with TIICs and immune checkpoint

By applying TIIC status in Cox model, we found that only macrophage infiltration score [TMR, XCL, CBS, QTS and MCP (Fig. 7L)] could indicate increased risk and be used as a poor prognostic predictor. However, other TIICs such as CD8^+^ T cell (Fig. 7K), CD4^+^ T cell, B cell, Neutrophil and NK cell didn’t have prognostic significance. We speculated whether ACLY and immune checkpoint related gene expression could be critical determinants influencing HCC prognosis. Consequently, decreased infiltration level of CD8^+^ T cell (KM curve: TMR: HR  =  0.421; p  =  0.024; MCP: HR  =  0.555; p  =  0.095), myeloid dendritic cell (KM curve: MCP: HR  =  0.432; p  =  0.051, XCL: HR  =  0.418; p  =  0.017; TMR: HR  =  0.508; p  =  0.064) and NK cell (KM curve: MCP: HR  =  0.420; p  =  0.026) was associated with poor overall survival in low ACLY expression group (Fig. 7M–O).

Meanwhile, decreased expression of CD276 accompanied by increased CD8  +  T cell had lower risk (KM curve: TMR: HR  =  0.430; p  =  0.027; CBS: HR  =  0.412; p  =  0.015; Fig. 7P, Q). Moreover, low expression of ACLY and CD276 with high infiltration level of CD8  +  T cell turned out to contribute to the best survival outcome (KM curve: TMR: HR  =  0.279; p  =  0.003; MCP: HR  =  0.479; p  =  0.060; CBS: HR  =  0.401; p  =  0.031; Fig. 7R, S) in HCC. The results might demonstrate the evidence of interaction between ACLY, TIICs and/or immune checkpoints in TME, suggesting that potential metabolism difference regulated by ACLY could play an unignorable role in immune function and consequently influenced prognosis in HCC.

### Establishment of a novel gene signature to predict HCC prognosis

To explore predictive markers, 4559 DEGs were enrolled and processed under VST transformation (Fig. 7A) in candidate gene group. One hundred nineteen kinds of TIICs estimated in six algorithms were enrolled in TIIC group. LASSO Cox regression model was fitted to build a predictive marker panel (Fig. 7B) in TCGA cohort. The lambda value chosen by ten-fold cross-validation was drawn at a vertical line (Fig. 7C). The Cox regression forest map served as a visualization of the correlation matrix (Fig. 7D). In TIIC group, no panel was found to show sufficient predictive power in Cox model (Additional file 15: Figure S15). In candidate gene group, seven genes were qualified through the multicollinearity test in secondary modeling. The seven genes were working together as a predictive gene panel, including CBX2 (p  =  0.111); LCAT (p  =  0.110); SPP1 (p  =  0.152); KPNA2 (p  =  0.040); GAGE4 (p  =  0.047); SOCS2 (p  =  0.019) and PGA4 (p  <  0.001). The detailed information of participants and HR were provided in Fig. 7E. The model was with a medium accuracy of predictive ability (concordance index  =  0.73). Through accumulation of individual risk score, total risk scores were calculated from the designed formula. Patients were divided into low-risk or high-risk group with the median risk score working as the cutoff value. AUC values were calculated from 1 to 5 years every 0.2 year and time-dependent ROC curves were drawn in Fig. 7F. The maximum AUC value occurred at 1.2 years (438 days). Furthermore, the gene panel in our model reflected significant predictive power in KM curves (Fig. 7G). We also compared the expression correlation between ACLY and the predictive gene panel in 40 kinds of tumors in published data (Fig. 7H). ACLY had association with CBX2 (Spearman’s rho  =  0.394; p  <  0.001), GAGE4 (Spearman’s rho  =  0.232; p  <  0.001), KPNA2 (Spearman’s rho  =  0.656; p  <  0.001), LCAT (Spearman’s rho  =  − 0.272; p  <  0.001) and SPP1 (Spearman’s rho  =  0.321; p  <  0.001) in HCC (Fig. 7I).

## Discussion

HCC is a malignancy with a distinct ability to invade and grow within the hepatic vasculature. Approximately 20% of patients have microvascular invasion (MVI) at the time of diagnosis. Among patients with unresectable tumors, the probability of developing portal vein tumor thrombosis (PVTT) at 1 and 3 years is 21 and 46%, respectively. The incidence of HCC accompanied by PVTT was 44–62.2%, with the median survival period without any interventions for 2.7 months only [2, 3], due to the fact that PVTT can lead to the wide dissemination of tumors throughout the liver and cause a marked deterioration of hepatic function. On one hand, MVI impairs liver function through reduced liver perfusion as a result of impaired blood flow, either directly via reduced inflow in PVTT or via elevated sinusoidal pressure in hepatic vein invasion. On the other hand, extension of tumor within the vasculature promotes tumor spread beyond the liver via direct seeding and extension. The impact on prognosis is thus profound and predictable.

Another important question is whether there exist progressive molecular alterations between primary tumors and matched PVTTs. Ye et al. [4] found that the gene expression patterns of metastatic lesions are nearly identical to their corresponding primary HCCs. Similar results have been observed for somatic mutations and miRNA expressions: Huang et al. [3] found that more than 94% somatic mutations are shared by primary tumor and PVTTs, and Wong et al. [6] reported that no obvious difference of miRNA expressions could be found between primary HCCs and the venous metastases. As these previous studies have declared, computational analysis shows that the inter-patient differences are much larger than the intra-patient heterogeneities between matched primary tumor and PVTT in most cases. Few consistent molecular alterations have be found between primary tumors and matched PVTTs. However, we have noticed that a few patients may undergo progressive molecular alterations according to the clustering analysis. Therefore, we used a novel individualized differential analysis strategy to identify the progressively differentially expressed genes between matched primary tumor and PVTT for each patient. Results showed that different patients were characterized with very different numbers of progressively differentially expressed genes.

The concept of metabolism reprogramming was first proposed a century ago, which is a sign of malignant tumors and plays a critical role in the process of tumor carcinogenesis [8, 47]. HCC is such a heterogeneous malignancy at metabolic level [46]. Our study indicated that there is an increase in fatty acid biosynthesis in both HCC tumor and PVTT samples by mass spectrometry analysis. Moreover, several core markers in fatty acid biosynthesis were upregulated through identification of GO, KEGG annotation by enrichment analysis, among which ACLY showed the most obvious alteration. ACLY is a cross-link representative between glucose and/or glutamine metabolism and fatty acid biosynthesis and/or mevalonate pathways, which plays an important role in various kinds of tumors. Under the pressure of insufficient energy supply from mitochondria and hypoxia, ACLY acts as a vital building block which provides acetyl coenzyme for intracellular biosynthesis of fatty acids and cholesterol [13, 48]. Gu’s study identified an IKKβ-USP30-ACLY axis that plays an essential role in the lipogenesis and liver cancer [49]. Han’s study indicated that knockdown of ACLY remarkably suppressed stemness properties, migration and invasion in HCC cells [50]. By identification of DEGs, functional and correlation analysis, considering the interaction of glucose and lipid metabolism connected by ACLY, we provided evidence that ACLY may work as a positive upstream regulator of fatty acid biosynthesis and activate a bypass of energy supplement besides Warburg effect in HCC, validating its significance function in metabolism.

The former results bring us to consider whether immune function is associated with ACLY and to seek meaningful clinical intervention targets. Immune-deficient tumors have decreased immune cell infiltration, and they are mainly manifested as highly proliferative tumor cells with high fatty acid metabolism or neuroendocrine function [51]. It has been recognized that most types of tumors without T cell infiltration are with a relatively poor prognosis and cannot benefit from immunotherapy [51, 52]. However, we haven’t explored a differential survival outcome with immune checkpoints and TIIC infiltration level except macrophage in TCGA HCC cohort. The phenomenon might be explained by aetiologic diversity and heterogeneous environmental factors of HCC compared with other solid tumors [45]. We then demonstrated the correlation between immune checkpoint, TIIC infiltration and ACLY. Trough stratification of ACLY transcriptomic level, the prognostic value of TIIC infiltration and immune checkpoint remarkably enhanced, indicating the underlying function of ACLY in HCC immune regulation and consequently influenced patients’ survival outcomes.

Given the metabolic imbalance of tumor cells, many drugs have shown good clinical application prospects and entered clinical trials. By negatively regulating the related pathways in metabolism, especially those that block the de novo synthesis of fat acid, molecule inhibitors targeting ACLY such as SB-204990, hydroxycitric acid and ECT-100 could inhibit the occurrence and development of HCC [53, 54]. HCC was a malignancy only partially benefit from immune-checkpoint therapy and strongly affected by the ongoing inflammation [55]. Anti-PD-1/PD-L1 antibodies have shown clinical advantages in more than 15 cancer types, but most patients with advanced HCC have not yet obtained clinical benefit from these drugs, which indicates that the immunosuppressive mechanism in the tumor microenvironment may of a great essence [56]. This begs the question that whether metabolism could interact with immune system and sensitize the anti-tumor function of immune therapy. To our surprise, however, there is almost no therapy targeting ACLY inhibition combined with immune therapy. Only Gu’s study recently elucidated that the combination of ACLY blockade ECT-1002 and PD-L1 antibodies can inhibit HCC tumorigenesis and progression [49]. In the current study, we further elucidated the prognostic effectiveness of combined expression of ACLY plus PD1, CTLA4 and potential immune checkpoints in clinical trials such as CD276.

As is summarized above, considering that the mechanism of single-gene ACLY in HCC has been clarified recently, we tried to emphasize on the prognostic value of ACLY and its interaction with immune microenvironment. We indeed found a novel immune checkpoint via transcriptomic analysis in clinical samples: CD276. In former studies, CD276 CAR-T cells showed potent antitumor activity against solid tumor cells [57]; a dual-compartment targeted CD276 ADC antibody reagent could simultaneously destroy both tumor cells and tumor vasculature [58]. The former model was based on CAR-T and the latter directly discussed the anti-tumor and anti-vasculature function of CD276 but not immune function. Enoblituzumab was the only investigational monoclonal antibody targeting CD276 which was evaluated in Phase 1 studies. The translational results of CD276 as a new immune checkpoint were still very limited. We hope that more works and drugs would be available in the future to validate the immune mechanism of CD276 and its association with ACLY.

There are certain limitations in our study. Both GEO and TCGA cohorts and samples we provided are analyzed retrospectively. More studies on mechanism through experimental consolidation and randomized controlled trials are required to further explore the translational value of the combination therapy of ACLY metabolic inhibitors and immune checkpoint blockades.

## Conclusions

In summary, we compared different sample types in HCC and found no difference between tumor and PVTT samples through several approaches. Then we further investigated a novel gene, ACLY, and its indispensable role in metabolism, immune function and a prognostic gene panel in HCC. Trough stratification of ACLY expression, our work reversed the insufficient prognostic value of immune checkpoints and TIIC infiltration, suggesting that the combination inhibition of ACLY and immune checkpoints may shed light on HCC treatment.

## Supplementary Information

**Additional file 1: **Supplementary Data 1A Huashan HCC Cohort clinical data and brief summary. Supplementary Data 1B TCGA HCC Cohort Clinical Data. Supplementary Data 2 gene list rank-ordered with enrichment score by signal2Noise in GSEA analysis. Supplementary Data 3A Whole transcriptomic expression estimation of different TIICs in GSE77509 cohort. Supplementary Data 3B Whole transcriptomic expression estimation of different TIICs in TCGA HCC cohort. Supplementary Data 4A Detailed results of multivariable Cox proportional hazard model of 20 genes in fatty acid biosynthesis process in 40 kinds of tumors in TCGA database. Supplementary Data 4B Detailed results of multivariable Cox proportional hazard model of 20 genes in cholesterol biosynthesis process in 40 kinds of tumors in TCGA database.

**Additional file 2: Figure S2.** DEGs in TCGA cohort and whole functional enrichment analysis of GSE 77509. (A) Volcano map showed all DEGs in TCGA. (B) KEGG annotation showed the enriched gene sets with the optimal FDR p value in GEO cohort. (C–H) The enriched gene sets were with the optimal FDR p value by GO annotation of cellular component, biological process and molecular function in GEO cohort. It is noteworthy that ATPase related activities were enriched in GO molecular function, which indicated the upregulation of energy consumption in HCC tumor tissue.

**Additional file 3: Figure S3.** Gene panels that had close interaction with metabolism and oncogenesis. (A) All enriched gene sets (n = 14) in KEGG annotation. (B) In addition to fatty acid metabolism, cell cycle, homologous recombination and Fanconi anemia pathway, were cited in gene sets.

**Additional file 4: Figure S4.** Members participated in fatty acid biosynthesis process (A) and mevalonate pathway (B) in TCGA cohort. Similarly, fatty acid synthesis was more activated in tumor tissue than cholesterol synthesis.

**Additional file 5: Figure S5.** Whole correlation analysis of ACLY, fatty acid biosynthesis process and cholesterol biosynthesis process in TCGA database. ACLY was positively correlated with both pathways in HCC and other malignancies in TCGA cohort. Solid squareness indicates the qualified p-value (p < 0.05) in analysis.

**Additional file 6: Figure S6.** ACLY interacted with immune checkpoints and their predictive impacts in TCGA cohort. CD276, FGL1, HAVCR2, KLRC1, LAG3, SIGLEC15 and TIGIT are targets under pre-clinical studies and clinical trials. (A) Integrated correlation analysis of ACLY and immune checkpoint signatures in TCGA database. (B–F) Detailed plots showing relevance between ACLY and promising immune checkpoints. (G–O) Survival consequence of patients classified by immune checkpoint expression (high risk group: > median expression value, low risk group: ≤ median expression value). FGL1, Fibrinogen-like Protein 1; HAVCR2, Hepatitis A Virus Cellular Receptor 2, encoding TIM-3; KLRC1, Killer Cell Lectin Like Receptor C1, encoding NKG2A; LAG3, Lymphocyte-activation Gene 3; SIGLEC15, Sialic Acid-binding Ig-like Lectin 15; TIGIT, T cell immunoreceptor with Ig and ITIM domains.

**Additional file 7: Figure S7.** TIIC estimation in 50 patients (50 tumor tissue samples and 50 homologous adjacent normal tissue samples in TCGA cohort) computed by six algorithms. CD8+ T cells and NK cells were escaped from HCC tumor samples, which are two TIICs considered inducing anti-tumor function.

**Additional file 8: Figure S8.** Immune landscape in an unsupervised hierarchical clustering view in GEO cohort (Figure 8) and TCGA cohort (Figure 9). Patients were stratified in four major subtypes: S1 (immune cell inflamed), S2 (immune cell escaped), S3 (immune desert) and a new subtype: S4 (macrophage/monocyte infiltrated). The former three subtypes have been reported by several studies. The mechanism of macrophage/monocyte infiltration in HCC as a unique subtype requires to be further investigated.

**Additional file 9: Figure S9.** Immune landscape in an unsupervised hierarchical clustering view in GEO cohort (Figure 8) and TCGA cohort (Figure 9). Patients were stratified in four major subtypes: S1 (immune cell inflamed), S2 (immune cell escaped), S3 (immune desert) and a new subtype: S4 (macrophage/monocyte infiltrated). The former three subtypes have been reported by several studies. The mechanism of macrophage/monocyte infiltration in HCC as a unique subtype requires to be further investigated.

**Additional file 10: Figure S10.** Differentially infiltrated immune cells estimated by MCP and EPC. There was no infiltration difference in tumor and PVTT. Furthermore, adjacent normal tissue turned out to have a higher level of immune cell infiltration.

**Additional file 11: Figure S11.** Relationships among ACLY expression level and TIIC level in TCGA cohort. Neutrophils and macrophages seemed to have an increased number of positive correlations in various kinds of malignancies. While infiltration level of NK cell and CD8+ T cell were negatively correlated with ACLY expression. This may explain the reason why high ACLY expression is associated with poor prognosis. Mechanism study is needed for further consolidation.

**Additional file 12: Figure S12.** Overall cell-type specific transcriptomic expression evaluated by CIBERSORTx in TCGA cohort. Targets such as metabolism associated genes and immune checkpoints were estimated in cell-type specific level to elucidate the difference between various TIICs and select ideal immune cell markers.

**Additional file 13: Figure S13.** T-SNE analysis for ACLY and CD276 expression subtypes. 361 TCGA HCC patient samples were enrolled to explore the stratification value of ACLY and CD276 through transcriptomic expression. The subjects are color-coded with high expression level (n = 180) and low expression level (n = 181).

**Additional file 14: Figure S14.** K-M curves and multivariate Cox model for lipid metabolism related gene signature defined in our study. (A–L) Apart from ACLY, there are many candidates qualified for prognosis prediction (overall survival and disease free survival), especially in fatty acid biosynthesis process. (M, N) Clinical information (age, sex, race, stage and tumor purity estimated by TIMER2.0) and gene expression matrix were included for Cox modeling. Tumor-type specific z score (normalization transformation processed) are illustrated in heatmap.

**Additional file 15: Figure S15.** LASSO Cox regression model demonstrated insufficient predict power for a panel of TIICs. Each label represents for a type of TIIC. (A) Correlation matrix revealed no multicollinearity among 4 TIICs. (B) Forest plot demonstrated HR and p value of multivariate Cox model enrolled TIICs. Each TIIC has enough power to predict survival outcome. (C) Time-dependent ROC curves of different time length for optimal observation time window. The best time window of the panel is still poor for prediction. (D) Kaplan–Meier survival analysis showed no difference between high and low infiltration group. There is also no qualified TIIC to establish a predictive model when DEGs are included. A69, CD4+ T cell Th2 XCELL; A70, B cell plasma CIBERSORT; A95, CD8+ T cell CIBERSORT; A101, Macrophage M0 CIBERSORT.

## Abbreviations

HCC: Hepatocellular carcinoma
PVTT: Portal vein tumor thrombosis
ACLY: ATP citrate lyase
AFP: Alpha-fetoprotein
BCLC: Barcelona Clinic Liver Cancer
CoA: Coenzyme A
PD-1: Programmed cell death protein 1
PD-L1: Programmed death 1 ligand 1
TME: Tumor microenvironment
GEO: Gene Expression Omnibus
TIICs: Tumor-infiltrating immune cells
DEGs: Differentially expressed genes
ANT: Adjacent normal tissue
DN: Distal noncancerous tissue
UHPLC-MS: Ultra-high-performance liquid chromatography-mass spectrometry
VST: Variance stabilizing transformation
FDR: False discovery rate
DEA: Differentially expressed gene analysis
GSEA: Gene set enrichment analysis
BP: Biological process
CC: Cellular component
MF: Molecular functional
GO: Gene Ontology
KEGG: Kyoto Encyclopedia of Genome and Genome
LASSO: Least absolute shrinkage and selection operator
t-SNE: T-distributed stochastic neighbor embedding
TMR: TIMER
CBS: CIBERSORT
QTS: QuanTIseq
XCL: XCell
MCP: MCP-counter
EPIC: EPC
VIF: Variance inflation factor
CV: Coefficient of variation
HR: Hazard ratio
MVI: Microvascular invasion

## References

[1] Bray F, Ferlay J, Soerjomataram I, Siegel RL, Torre LA, Jemal A (2018). Global cancer statistics 2018: GLOBOCAN estimates of incidence and mortality worldwide for 36 cancers in 185 countries. CA Cancer J Clin.

[2] Quirk M, Kim YH, Saab S, Lee EW (2015). Management of hepatocellular carcinoma with portal vein thrombosis. World J Gastroenterol.

[3] Pirisi M, Avellini C, Fabris C, Scott C, Bardus P, Soardo G, Beltrami CA, Bartoli E (1998). Portal vein thrombosis in hepatocellular carcinoma: age and sex distribution in an autopsy study. J Cancer Res Clin Oncol.

[4] Heimbach JK, Kulik LM, Finn RS, Sirlin CB, Abecassis MM, Roberts LR, Zhu AX, Murad MH, Marrero JA (2018). AASLD guidelines for the treatment of hepatocellular carcinoma. Hepatology.

[5] Forner A, Reig ME, de Lope CR, Bruix J (2010). Current strategy for staging and treatment: the BCLC update and future prospects. Semin Liver Dis.

[6] Connolly GC, Chen R, Hyrien O, Mantry P, Bozorgzadeh A, Abt P, Khorana AA (2008). Incidence, risk factors and consequences of portal vein and systemic thromboses in hepatocellular carcinoma. Thromb Res.

[7] Llovet JM, Zucman-Rossi J, Pikarsky E, Sangro B, Schwartz M, Sherman M, Gores G (2016). Hepatocellular carcinoma. Nat Rev Dis Primers.

[8] Koundouros N, Poulogiannis G (2020). Reprogramming of fatty acid metabolism in cancer. Br J Cancer.

[9] Hanahan D, Weinberg RA (2011). Hallmarks of cancer: the next generation. Cell.

[10] Liberti MV, Locasale JW (2016). The Warburg effect: how does it benefit cancer cells?. Trends Biochem Sci.

[11] Baenke F, Peck B, Miess H, Schulze A (2013). Hooked on fat: the role of lipid synthesis in cancer metabolism and tumour development. Dis Model Mech.

[12] Notarnicola M, Tutino V, Caruso MG (2014). Tumor-induced alterations in lipid metabolism. Curr Med Chem.

[13] Rohrig F, Schulze A (2016). The multifaceted roles of fatty acid synthesis in cancer. Nat Rev Cancer.

[14] Chypre M, Zaidi N, Smans K (2012). ATP-citrate lyase: a mini-review. Biochem Biophys Res Commun.

[15] Zhou Y, Bollu LR, Tozzi F, Ye X, Bhattacharya R, Gao G, Dupre E, Xia L, Lu J, Fan F (2013). ATP citrate lyase mediates resistance of colorectal cancer cells to SN38. Mol Cancer Ther.

[16] Qian X, Hu J, Zhao J, Chen H (2015). ATP citrate lyase expression is associated with advanced stage and prognosis in gastric adenocarcinoma. Int J Clin Exp Med.

[17] Zhang C, Liu J, Huang G, Zhao Y, Yue X, Wu H, Li J, Zhu J, Shen Z, Haffty BG (2016). Cullin3-KLHL25 ubiquitin ligase targets ACLY for degradation to inhibit lipid synthesis and tumor progression. Genes Dev.

[18] Hatzivassiliou G, Zhao F, Bauer DE, Andreadis C, Shaw AN, Dhanak D, Hingorani SR, Tuveson DA, Thompson CB (2005). ATP citrate lyase inhibition can suppress tumor cell growth. Cancer Cell.

[19] Khwairakpam AD, Shyamananda MS, Sailo BL, Rathnakaram SR, Padmavathi G, Kotoky J, Kunnumakkara AB (2015). ATP citrate lyase (ACLY): a promising target for cancer prevention and treatment. Curr Drug Targets.

[20] Gong J, Chehrazi-Raffle A, Reddi S, Salgia R (2018). Development of PD-1 and PD-L1 inhibitors as a form of cancer immunotherapy: a comprehensive review of registration trials and future considerations. J Immunother Cancer.

[21] Gandini S, Massi D, Mandala M (2016). PD-L1 expression in cancer patients receiving anti PD-1/PD-L1 antibodies: a systematic review and meta-analysis. Crit Rev Oncol Hematol.

[22] O'Donnell JS, Long GV, Scolyer RA, Teng MW, Smyth MJ (2017). Resistance to PD1/PDL1 checkpoint inhibition. Cancer Treat Rev.

[23] Jung J, Zeng H, Horng T (2019). Metabolism as a guiding force for immunity. Nat Cell Biol.

[24] Zheng FJ, Zhao XJ, Zeng ZD, Wang LC, Lv WJ, Wang QQ, Xu GW (2020). Development of a plasma pseudotargeted metabolomics method based on ultra-high-performance liquid chromatography-mass spectrometry. Nat Protoc.

[25] Smith CA, Want EJ, O'Maille G, Abagyan R, Siuzdak G (2006). XCMS: processing mass spectrometry data for metabolite profiling using nonlinear peak alignment, matching, and identification. Anal Chem.

[26] Kuhl C, Tautenhahn R, Bottcher C, Larson TR, Neumann S (2012). CAMERA: an integrated strategy for compound spectra extraction and annotation of liquid chromatography/mass spectrometry data sets. Anal Chem.

[27] Kessner D, Chambers M, Burke R, Agusand D, Mallick P (2008). ProteoWizard: open source software for rapid proteomics tools development. Bioinformatics.

[28] Love MI, Huber W, Anders S (2014). Moderated estimation of fold change and dispersion for RNA-seq data with DESeq2. Genome Biol.

[29] Yu GC, Wang LG, Han YY, He QY (2012). clusterProfiler: an R package for comparing biological themes among gene clusters. OMICS.

[30] Ginestet C (2011). ggplot2: elegant graphics for data analysis. J R Stat Soc A Stat.

[31] Subramanian A, Tamayo P, Mootha VK, Mukherjee S, Ebert BL, Gillette MA, Paulovich A, Pomeroy SL, Golub TR, Lander ES (2005). Gene set enrichment analysis: a knowledge-based approach for interpreting genome-wide expression profiles. P Natl Acad Sci USA.

[32] Bardou P, Mariette J, Escudie F, Djemiel C, Klopp C (2014). jvenn: an interactive Venn diagram viewer. BMC Bioinform.

[33] Uhlen M, Fagerberg L, Hallstrom BM, Lindskog C, Oksvold P, Mardinoglu A, Sivertsson A, Kampf C, Sjostedt E, Asplund A (2015). Tissue-based map of the human proteome. Science.

[34] Szklarczyk D, Gable AL, Lyon D, Junge A, Wyder S, Huerta-Cepas J, Simonovic M, Doncheva NT, Morris JH, Bork P (2019). STRING v11: protein-protein association networks with increased coverage, supporting functional discovery in genome-wide experimental datasets. Nucleic Acids Res.

[35] Li TW, Fu JX, Zeng ZX, Cohen D, Li J, Chen QM, Li B, Liu XS (2020). TIMER2.0 for analysis of tumor-infiltrating immune cells. Nucleic Acids Res.

[36] Li TW, Fan JY, Wang BB, Traugh N, Chen QM, Liu JS, Li B, Liu XS (2017). TIMER: a web server for comprehensive analysis of tumor-infiltrating immune cells. Cancer Res.

[37] Newman AM, Liu CL, Green MR, Gentles AJ, Feng WG, Xu Y, Hoang CD, Diehn M, Alizadeh AA (2015). Robust enumeration of cell subsets from tissue expression profiles. Nat Methods.

[38] Finotello F, Mayer C, Plattner C, Laschober G, Rieder D, Hackl H, Krogsdam A, Loncova Z, Posch W, Wilflingseder D (2019). Molecular and pharmacological modulators of the tumor immune contexture revealed by deconvolution of RNA-seq data. Genome Med.

[39] Aran D, Hu ZC, Butte AJ (2017). xCell: digitally portraying the tissue cellular heterogeneity landscape. Genome Biol.

[40] Becht E, Giraldo NA, Lacroix L, Buttard B, Elarouci N, Petitprez F, Selves J, Laurent-Puig P, Sautes-Fridman C, Fridman WH (2016). Estimating the population abundance of tissue-infiltrating immune and stromal cell populations using gene expression. Genome Biol.

[41] Racle J, de Jonge K, Baumgaertner P, Speiser DE, Gfeller D (2017). Simultaneous enumeration of cancer and immune cell types from bulk tumor gene expression data. Elife.

[42] Newman AM, Steen CB, Liu CL, Gentles AJ, Chaudhuri AA, Scherer F, Khodadoust MS, Esfahani MS, Luca BA, Steiner D (2019). Determining cell type abundance and expression from bulk tissues with digital cytometry. Nat Biotechnol.

[43] Moon SH, Huang CH, Houlihan SL, Regunath K, Freed-Pastor WA, Morris JP, Tschaharganeh DF, Kastenhuber ER, Barsotti AM, Culp-Hill R (2019). p53 represses the Mevalonate pathway to mediate tumor suppression. Cell.

[44] Sia D, Jiao Y, Martinez-Quetglas I, Kuchuk O, Villacorta-Martin C, de Moura MC, Putra J, Camprecios G, Bassaganyas L, Akers N (2017). Identification of an immune-specific class of hepatocellular carcinoma, based on molecular features. Gastroenterology.

[45] Hou JJ, Zhang HY, Sun BC, Karin M (2020). The immunobiology of hepatocellular carcinoma in humans and mice: basic concepts and therapeutic implications. J Hepatol.

[46] Calderaro J, Ziol M, Paradis V, Zucman-Rossi J (2019). Molecular and histological correlations in liver cancer. J Hepatol.

[47] Pavlova NN, Thompson CB (2016). The emerging hallmarks of cancer metabolism. Cell Metab.

[48] Zaidi N, Swinnen JV, Smans K (2012). ATP-citrate lyase: a key player in cancer metabolism. Cancer Res.

[49] Gu L, Zhu Y, Lin X, Lu B, Zhou X, Zhou F, Zhao Q, Prochownik EV, Li Y (2020). The IKKbeta-USP30-ACLY axis controls lipogenesis and tumorigenesis. Hepatology.

[50] Han Q, Chen CA, Yang W, Liang D, Lv HW, Lv GS, Zong QN, Wang HY (2020). ATP-citrate lyase regulates stemness and metastasis in hepatocellular carcinoma via the Wnt/beta-catenin signaling pathway. Hepatobiliary Pancreat Dis Int.

[51] Hegde PS, Chen DS (2020). Top 10 challenges in cancer immunotherapy. Immunity.

[52] Hegde PS, Karanikas V, Evers S (2016). The where, the when, and the how of immune monitoring for cancer immunotherapies in the era of checkpoint inhibition. Clin Cancer Res.

[53] Wei J, Leit S, Kuai J, Therrien E, Rafi S, Harwood HJ, DeLaBarre B, Tong L (2019). An allosteric mechanism for potent inhibition of human ATP-citrate lyase. Nature.

[54] Granchi C (2018). ATP citrate lyase (ACLY) inhibitors: an anti-cancer strategy at the crossroads of glucose and lipid metabolism. Eur J Med Chem.

[55] Shalapour S, Karin M (2019). Pas de deux: control of anti-tumor immunity by cancer-associated inflammation. Immunity.

[56] de Miguel M, Calvo E (2020). Clinical challenges of immune checkpoint inhibitors. Cancer Cell.

[57] Du H, Hirabayashi K, Ahn S, Kren NP, Montgomery SA, Wang X, Tiruthani K, Mirlekar B, Michaud D, Greene K (2019). Antitumor responses in the absence of toxicity in solid tumors by targeting B7–H3 via chimeric antigen receptor T cells. Cancer Cell.

[58] Seaman S, Zhu Z, Saha S, Zhang XM, Yang MY, Hilton MB, Morris K, Szot C, Morris H, Swing DA (2017). Eradication of tumors through simultaneous ablation of CD276/B7-H3-positive tumor cells and tumor vasculature. Cancer Cell.

