# Supplementary figures and images for: Identification and integrative analysis of ACLY and related gene panels associated with immune microenvironment reveal prognostic significance in hepatocellular carcinoma
Source: Cancer Cell Int. 2021 Aug 3;21:409. doi: 10.1186/s12935-021-02108-2 (PMC8335999; doi:10.1186/s12935-021-02108-2)

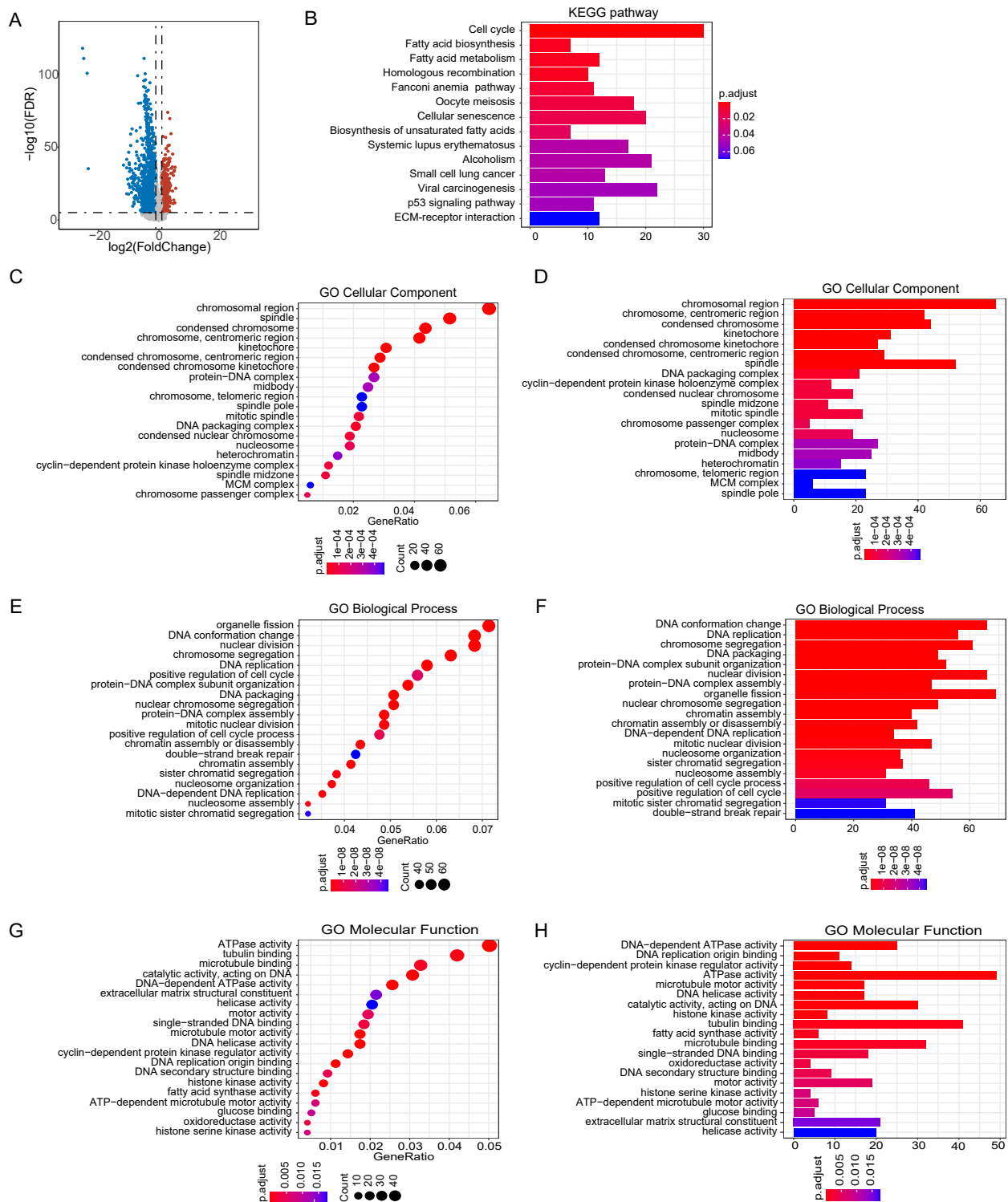

Supplementary Figure 2

Supplement: Supplementary file 2 — Additional file 2: Figure S2. DEGs in TCGA cohort and whole functional enrichment analysis of GSE 77509. (A) Volcano map showed all DEGs in TCGA. (B) KEGG annotation showed the enriched gene sets with the optimal FDR p value in GEO cohort. (C–H) The enriched gene sets were with the optimal FDR p value by GO annotation of cellular component, biological process and molecular function in GEO cohort. It is noteworthy that ATPase related activities were enriched in GO molecular function, which indicated the upregulation of energy consumption in HCC tumor tissue. [file 12935_2021_2108_MOESM2_ESM.pdf]

A

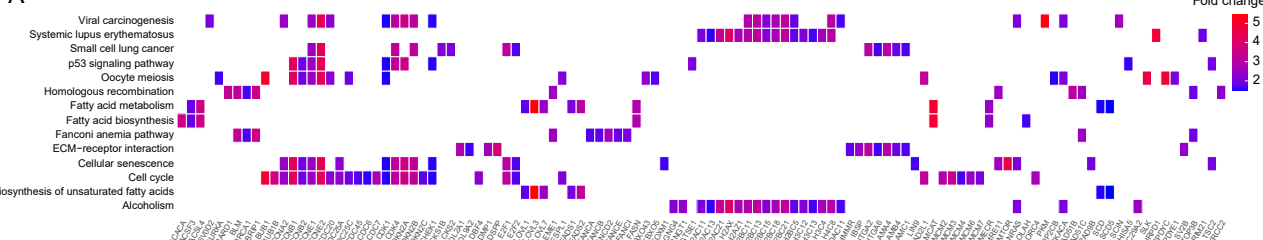

B

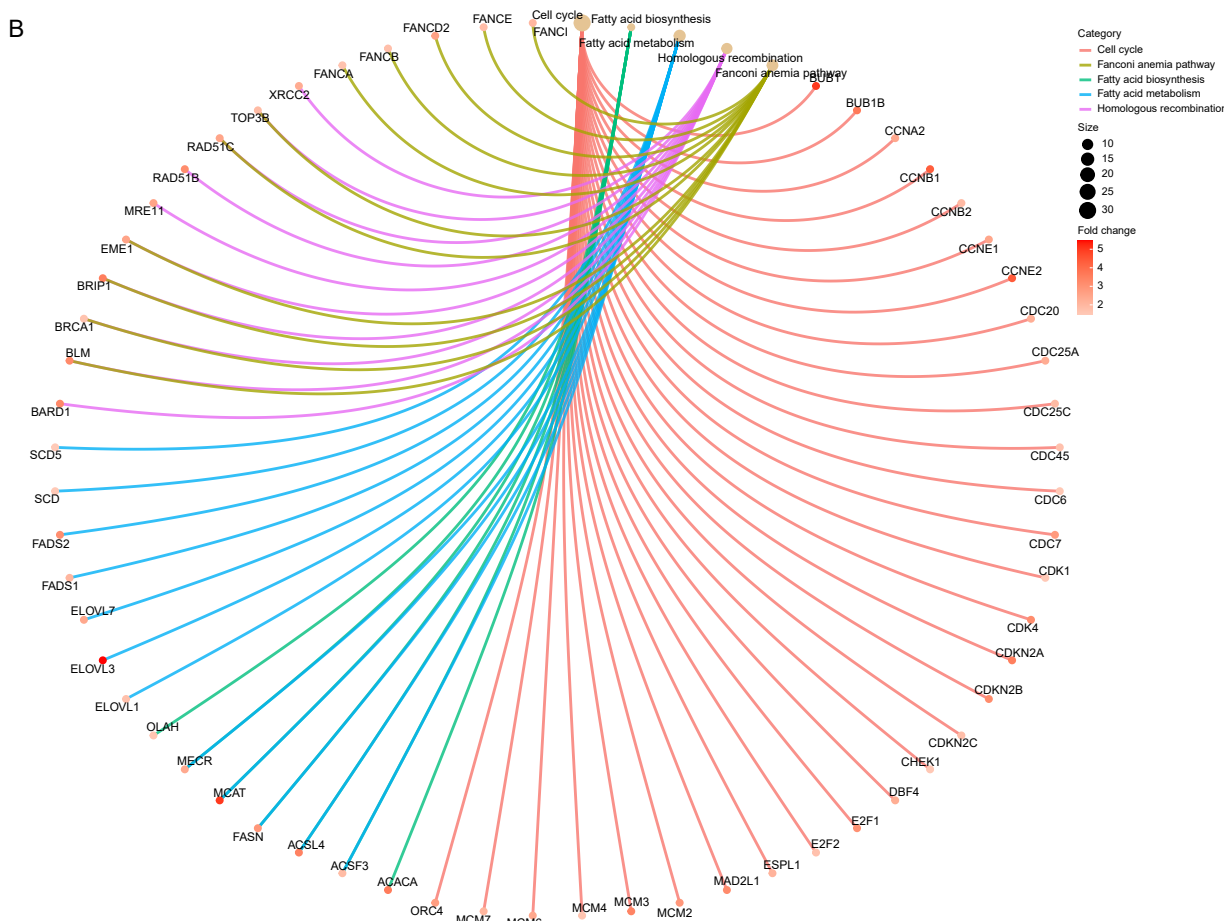

Supplementary Figure 3

Supplement: Supplementary file 3 — Additional file 3: Figure S3. Gene panels that had close interaction with metabolism and oncogenesis. (A) All enriched gene sets (n = 14) in KEGG annotation. (B) In addition to fatty acid metabolism, cell cycle, homologous recombination and Fanconi anemia pathway, were cited in gene sets. [file 12935_2021_2108_MOESM3_ESM.pdf]

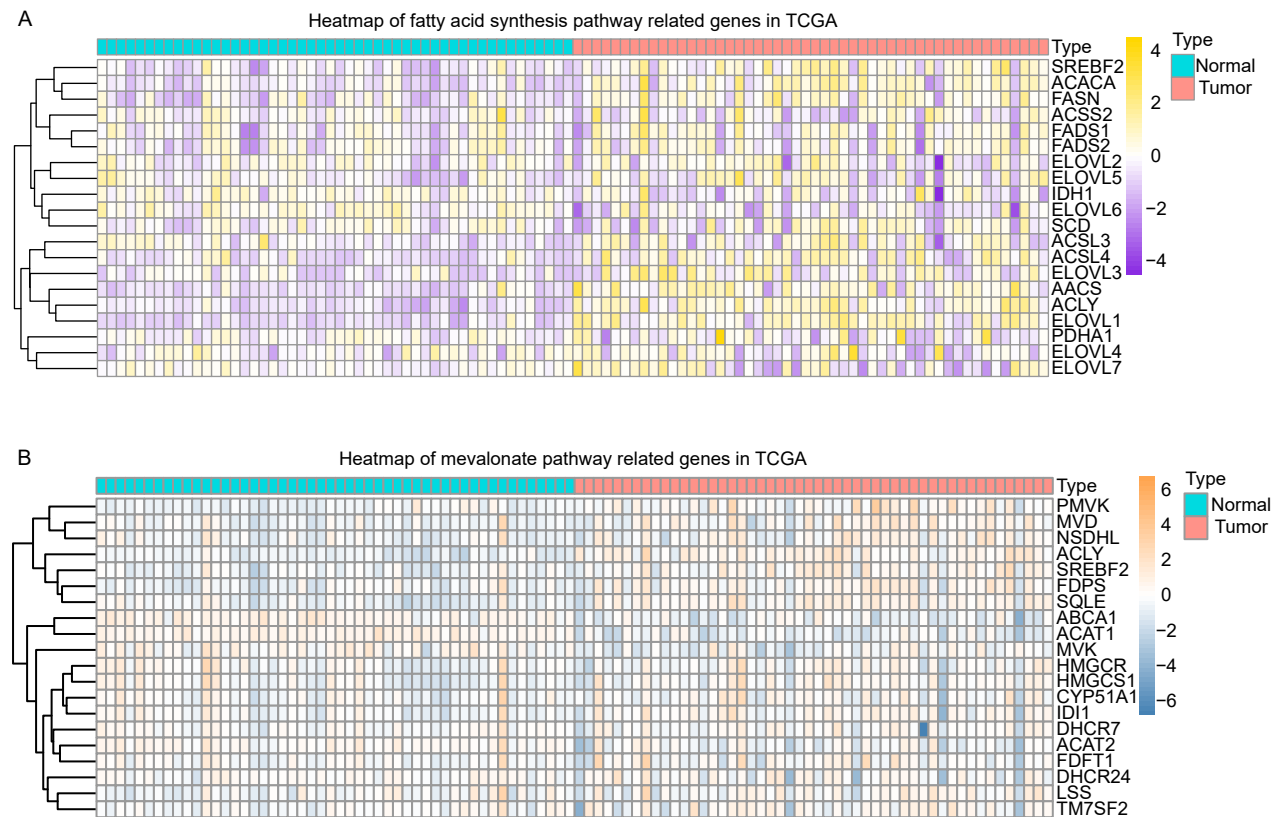

Supplementary Figure 4

Supplement: Supplementary file 4 — Additional file 4: Figure S4. Members participated in fatty acid biosynthesis process (A) and mevalonate pathway (B) in TCGA cohort. Similarly, fatty acid synthesis was more activated in tumor tissue than cholesterol synthesis. [file 12935_2021_2108_MOESM4_ESM.pdf]

A

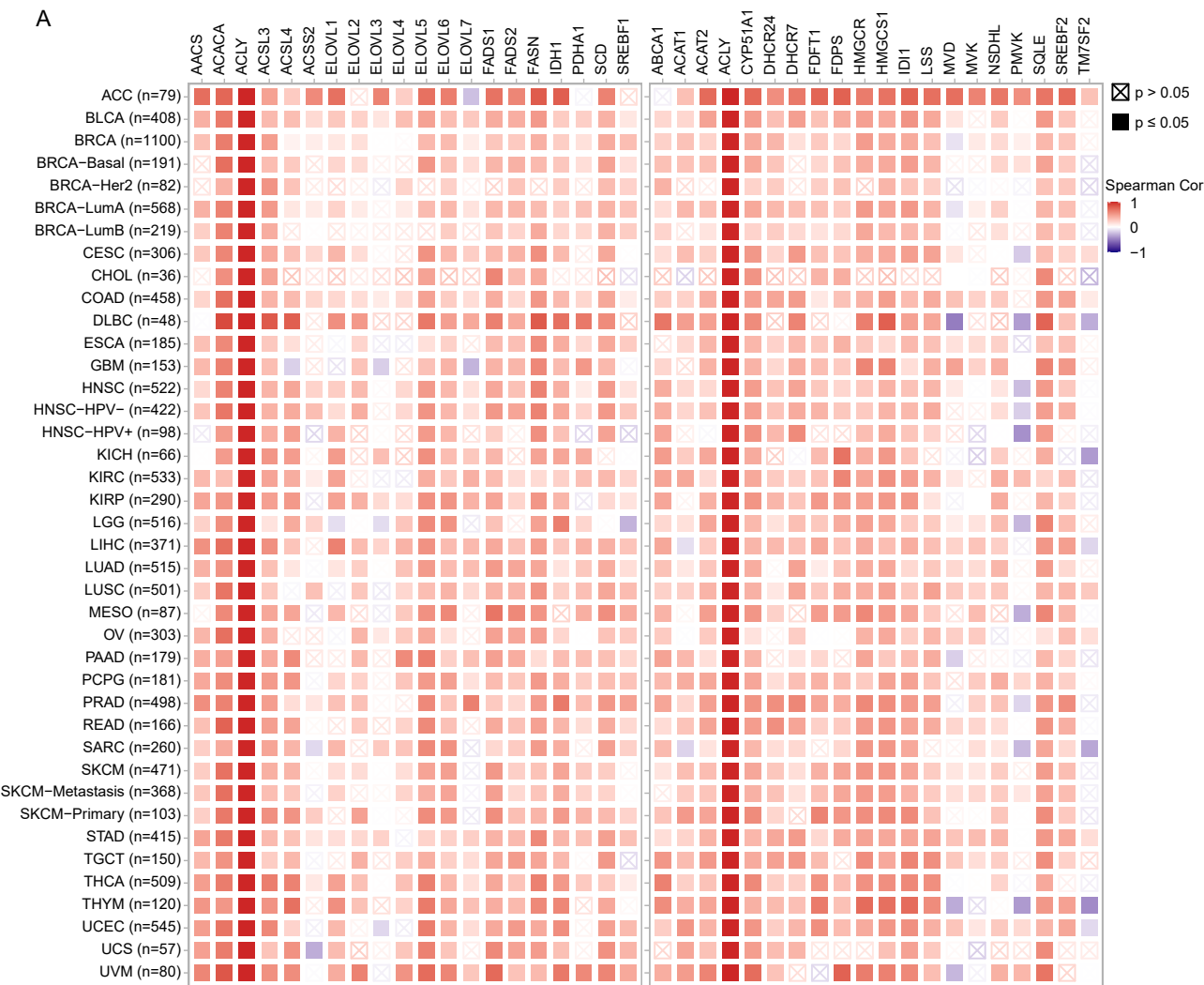

Supplementary Figure 5

Supplement: Supplementary file 5 — Additional file 5: Figure S5. Whole correlation analysis of ACLY, fatty acid biosynthesis process and cholesterol biosynthesis process in TCGA database. ACLY was positively correlated with both pathways in HCC and other malignancies in TCGA cohort. Solid squareness indicates the qualified p-value (p < 0.05) in analysis. [file 12935_2021_2108_MOESM5_ESM.pdf]

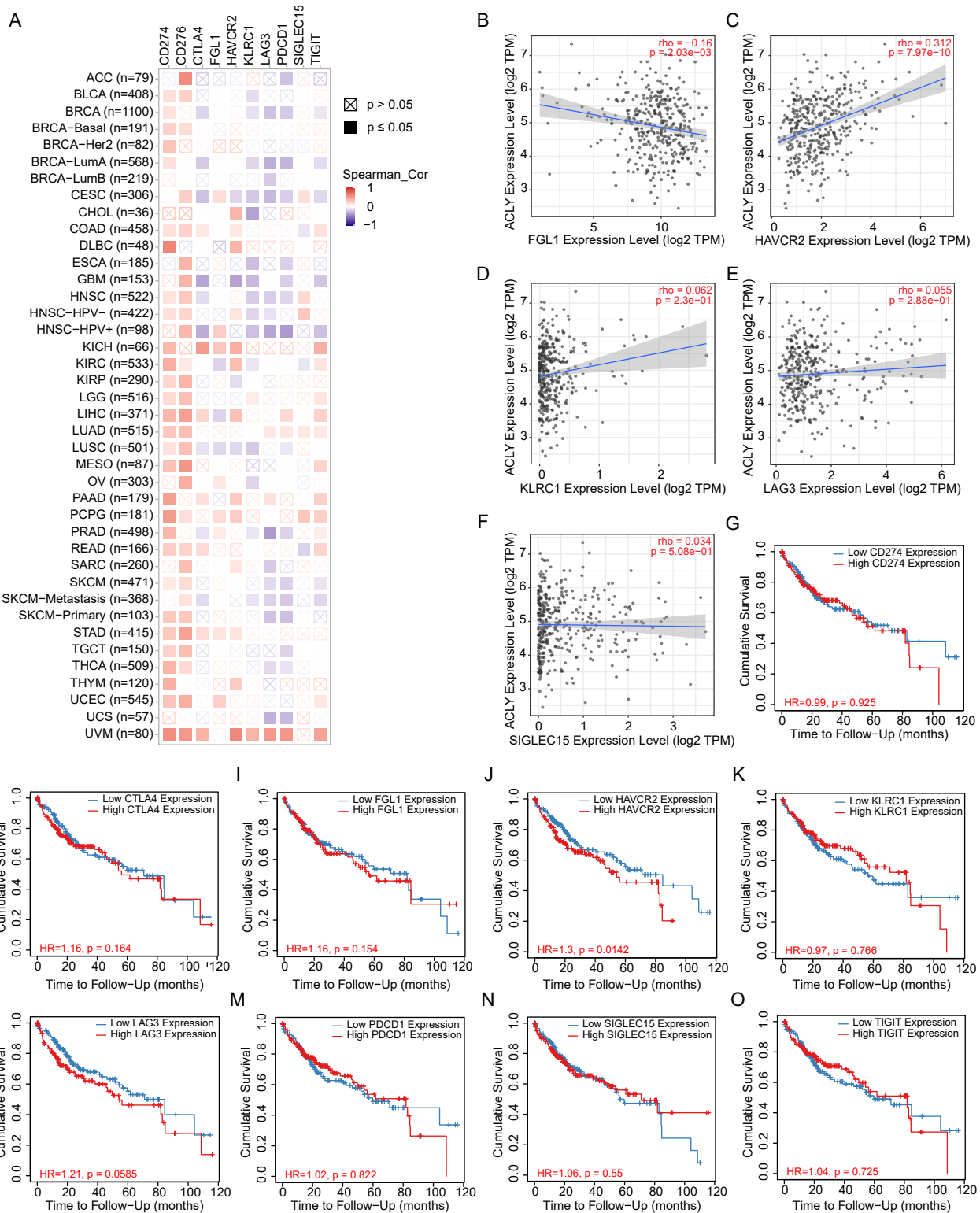

Supplementary Figure 6

Supplement: Supplementary file 6 — Additional file 6: Figure S6. ACLY interacted with immune checkpoints and their predictive impacts in TCGA cohort. CD276, FGL1, HAVCR2, KLRC1, LAG3, SIGLEC15 and TIGIT are targets under pre-clinical studies and clinical trials. (A) Integrated correlation analysis of ACLY and immune checkpoint signatures in TCGA database. (B–F) Detailed plots showing relevance between ACLY and promising immune checkpoints. (G–O) Survival consequence of patients classified by immune checkpoint expression (high risk group: > median expression value, low risk group: ≤ median expression value). FGL1, Fibrinogen-like Protein 1; HAVCR2, Hepatitis A Virus Cellular Receptor 2, encoding TIM-3; KLRC1, Killer Cell Lectin Like Receptor C1, encoding NKG2A; LAG3, Lymphocyte-activation Gene 3; SIGLEC15, Sialic Acid-binding Ig-like Lectin 15; TIGIT, T cell immunoreceptor with Ig and ITIM domains. [file 12935_2021_2108_MOESM6_ESM.pdf]

A

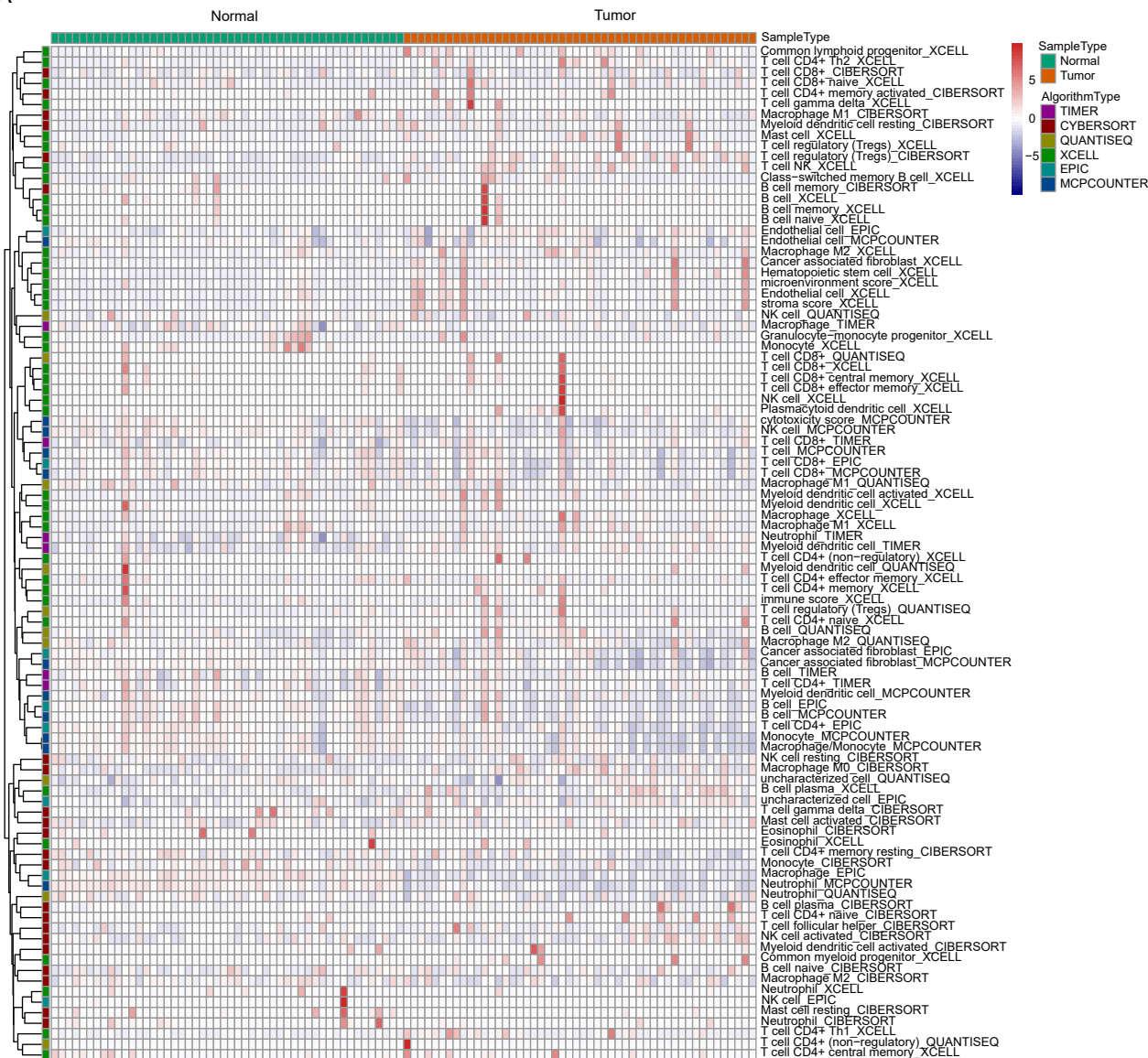

Supplementary Figure 7

Supplement: Supplementary file 7 — Additional file 7: Figure S7. TIIC estimation in 50 patients (50 tumor tissue samples and 50 homologous adjacent normal tissue samples in TCGA cohort) computed by six algorithms. CD8+ T cells and NK cells were escaped from HCC tumor samples, which are two TIICs considered inducing anti-tumor function. [file 12935_2021_2108_MOESM7_ESM.pdf]

A

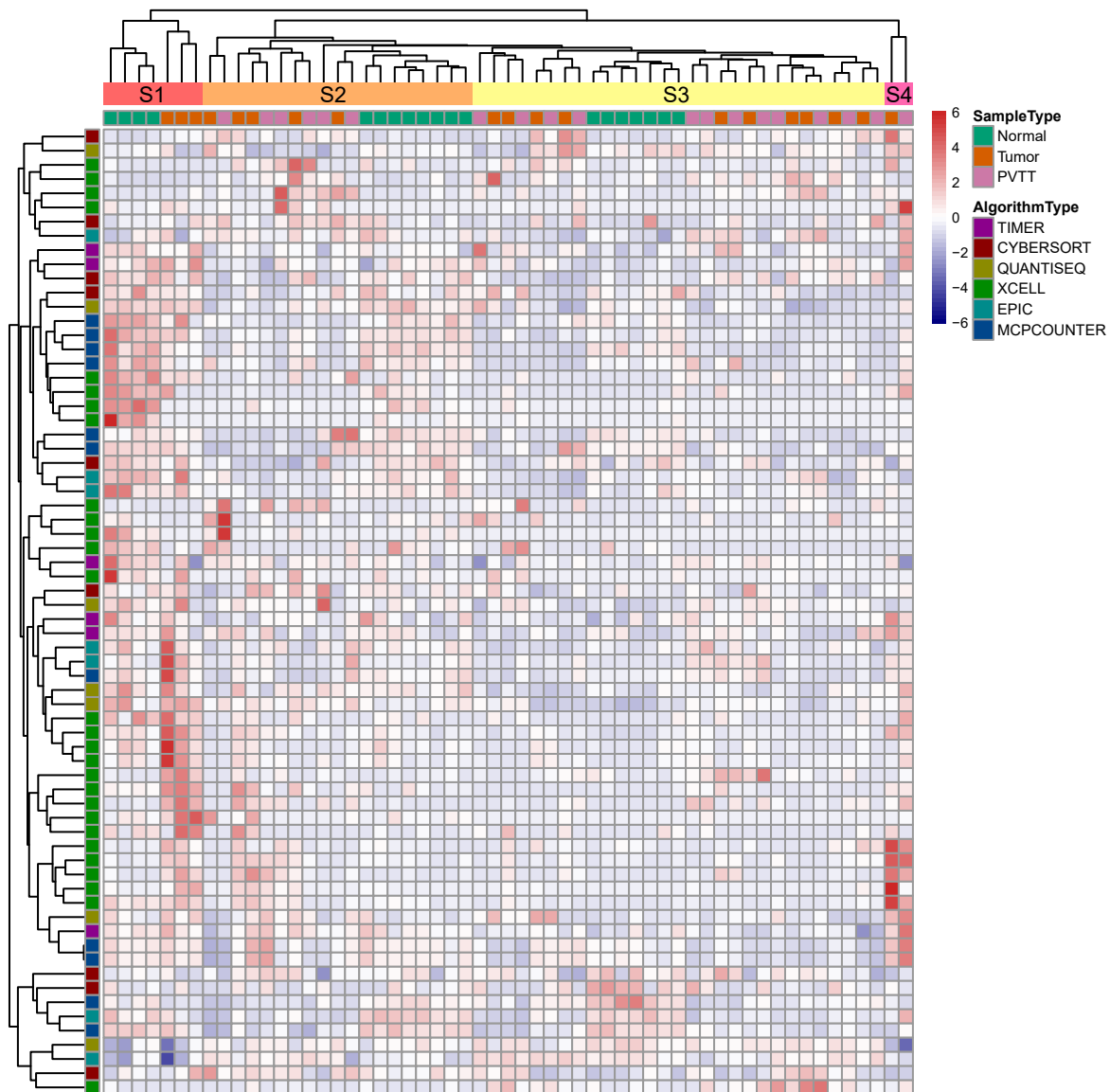

Supplementary Figure 8

Supplement: Supplementary file 8 — Additional file 8: Figure S8. Immune landscape in an unsupervised hierarchical clustering view in GEO cohort (Figure 8) and TCGA cohort (Figure 9). Patients were stratified in four major subtypes: S1 (immune cell inflamed), S2 (immune cell escaped), S3 (immune desert) and a new subtype: S4 (macrophage/monocyte infiltrated). The former three subtypes have been reported by several studies. The mechanism of macrophage/monocyte infiltration in HCC as a unique subtype requires to be further investigated. [file 12935_2021_2108_MOESM8_ESM.pdf]

A

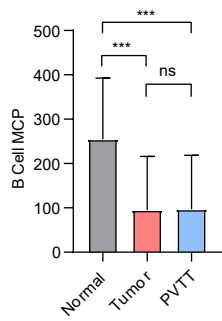

B

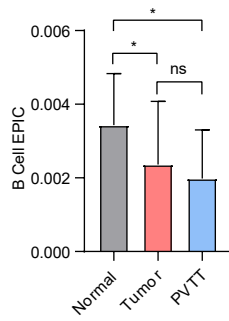

C

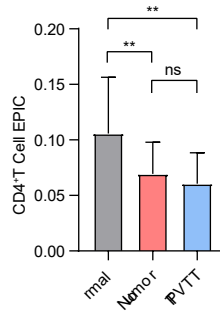

D

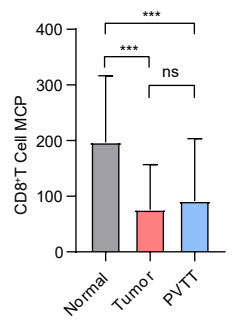

E

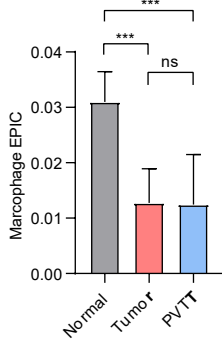

F

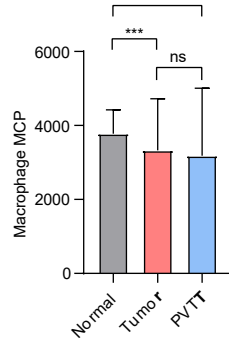

G

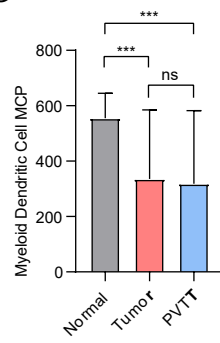

H

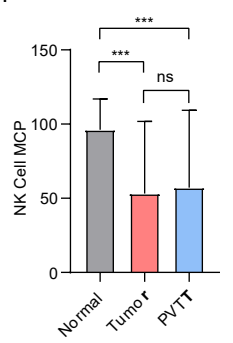

Supplementary Figure 10

Supplement: Supplementary file 10 — Additional file 10: Figure S10. Differentially infiltrated immune cells estimated by MCP and EPC. There was no infiltration difference in tumor and PVTT. Furthermore, adjacent normal tissue turned out to have a higher level of immune cell infiltration. [file 12935_2021_2108_MOESM10_ESM.pdf]

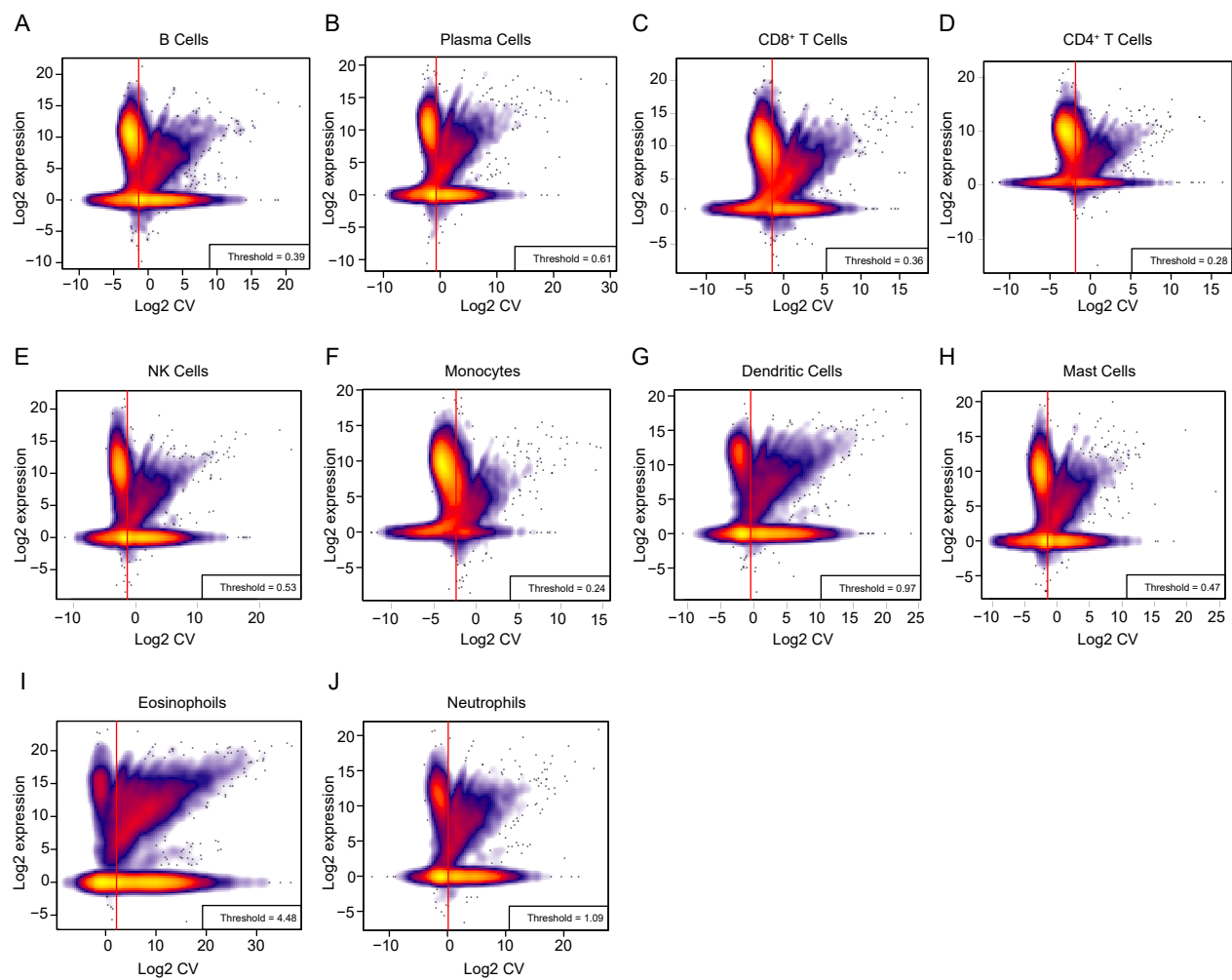

Supplementary Figure 12

Supplement: Supplementary file 12 — Additional file 12: Figure S12. Overall cell-type specific transcriptomic expression evaluated by CIBERSORTx in TCGA cohort. Targets such as metabolism associated genes and immune checkpoints were estimated in cell-type specific level to elucidate the difference between various TIICs and select ideal immune cell markers. [file 12935_2021_2108_MOESM12_ESM.pdf]

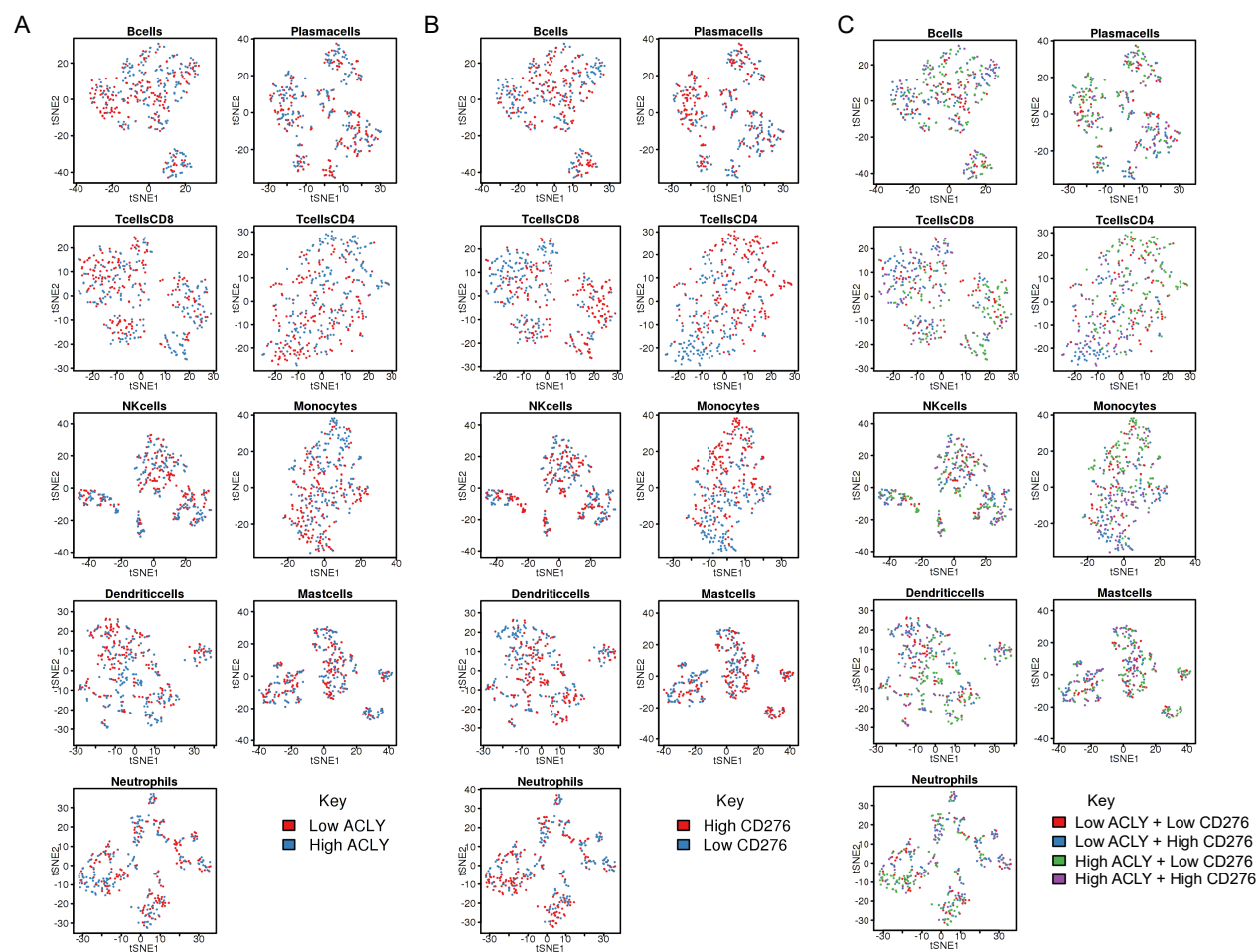

Supplementary Figure 13

Supplement: Supplementary file 13 — Additional file 13: Figure S13. T-SNE analysis for ACLY and CD276 expression subtypes. 361 TCGA HCC patient samples were enrolled to explore the stratification value of ACLY and CD276 through transcriptomic expression. The subjects are color-coded with high expression level (n = 180) and low expression level (n = 181). [file 12935_2021_2108_MOESM13_ESM.pdf]

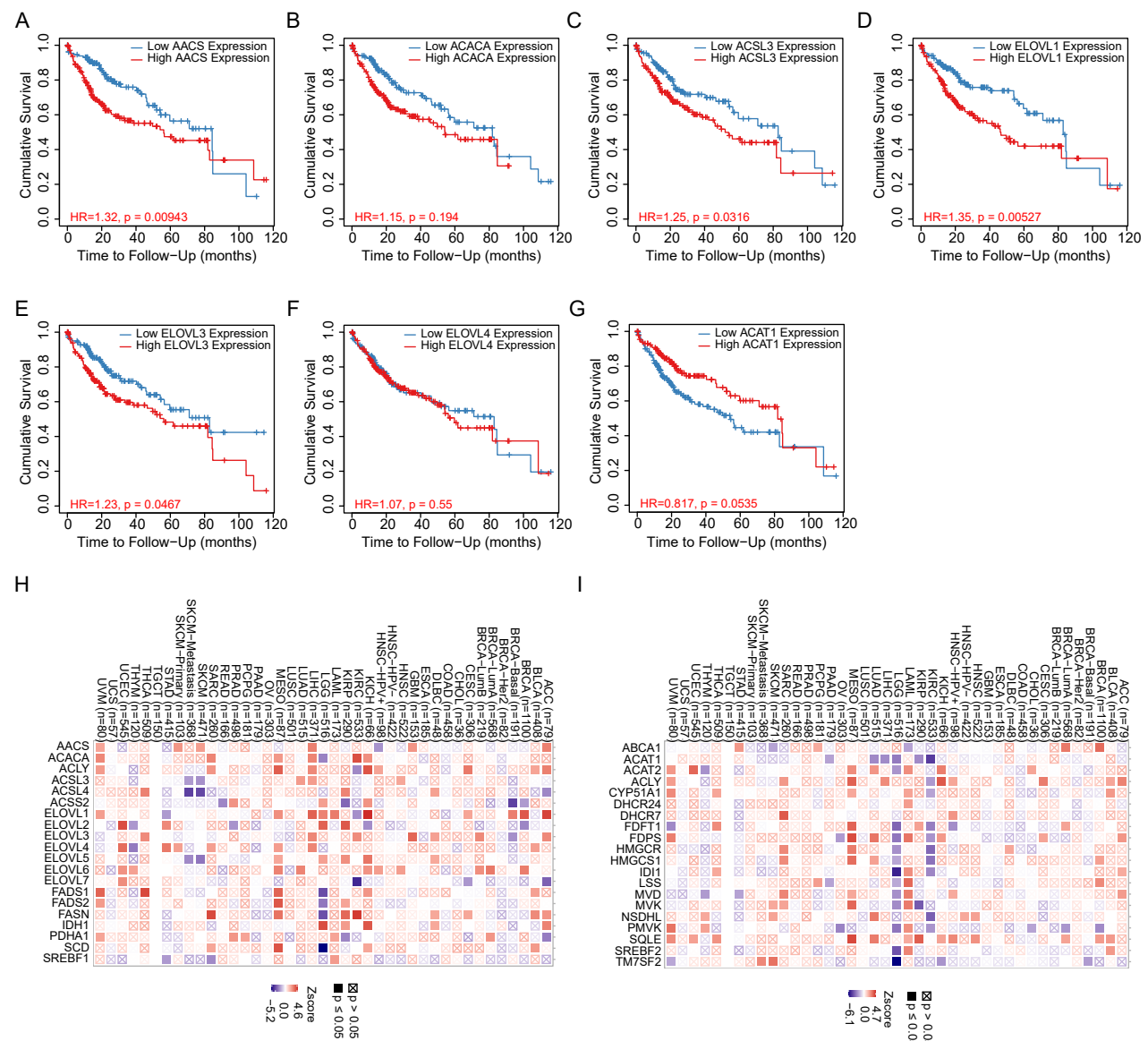

Supplementary Figure 14

Supplement: Supplementary file 14 — Additional file 14: Figure S14. K-M curves and multivariate Cox model for lipid metabolism related gene signature defined in our study. (A–L) Apart from ACLY, there are many candidates qualified for prognosis prediction (overall survival and disease free survival), especially in fatty acid biosynthesis process. (M, N) Clinical information (age, sex, race, stage and tumor purity estimated by TIMER2.0) and gene expression matrix were included for Cox modeling. Tumor-type specific z score (normalization transformation processed) are illustrated in heatmap. [file 12935_2021_2108_MOESM14_ESM.pdf]

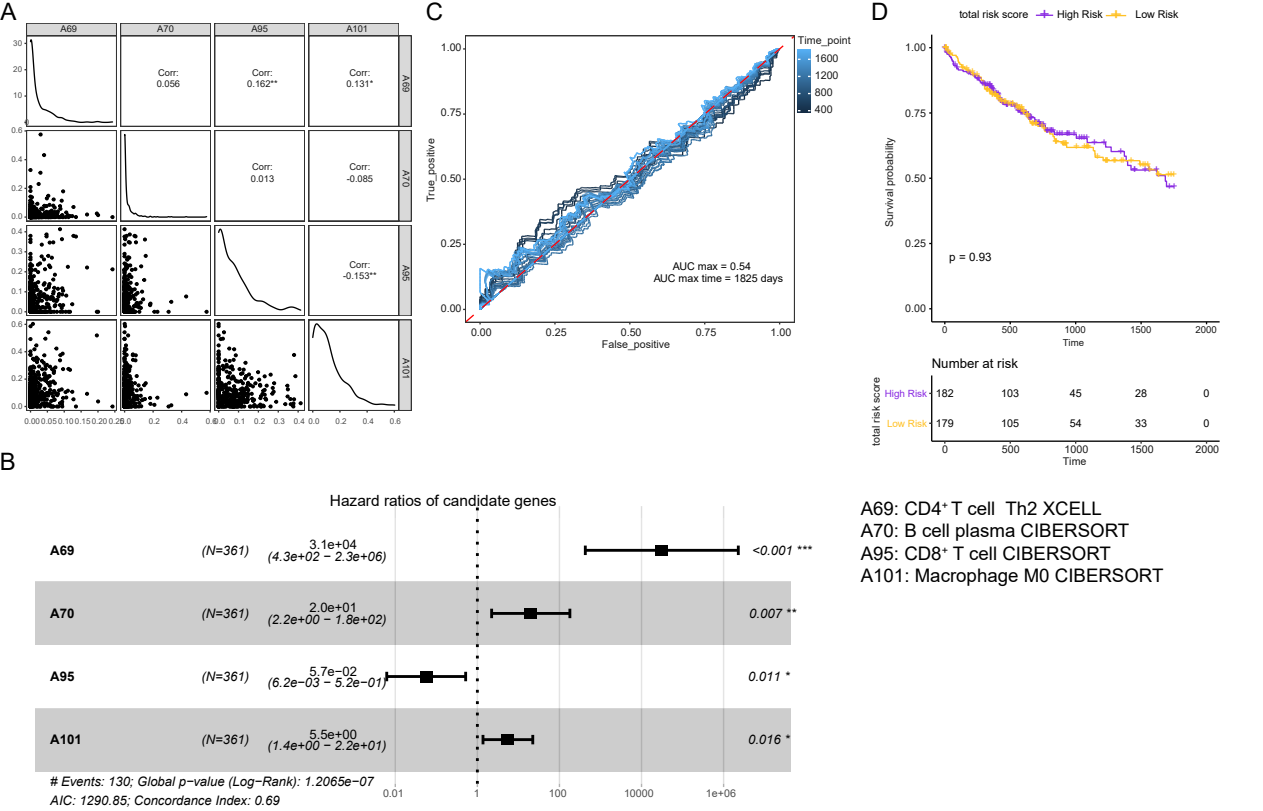

Supplementary Figure 15

Supplement: Supplementary file 15 — Additional file 15: Figure S15. LASSO Cox regression model demonstrated insufficient predict power for a panel of TIICs. Each label represents for a type of TIIC. (A) Correlation matrix revealed no multicollinearity among 4 TIICs. (B) Forest plot demonstrated HR and p value of multivariate Cox model enrolled TIICs. Each TIIC has enough power to predict survival outcome. (C) Time-dependent ROC curves of different time length for optimal observation time window. The best time window of the panel is still poor for prediction. (D) Kaplan–Meier survival analysis showed no difference between high and low infiltration group. There is also no qualified TIIC to establish a predictive model when DEGs are included. A69, CD4+ T cell Th2 XCELL; A70, B cell plasma CIBERSORT; A95, CD8+ T cell CIBERSORT; A101, Macrophage M0 CIBERSORT. [file 12935_2021_2108_MOESM15_ESM.pdf]
