# Supplementary material for: Identification and integrative analysis of ACLY and related gene panels associated with immune microenvironment reveal prognostic significance in hepatocellular carcinoma
Source: Cancer Cell Int. 2021 Aug 3;21:409. doi: 10.1186/s12935-021-02108-2 (PMC8335999; doi:10.1186/s12935-021-02108-2)
Supplement: Supplementary file 9 — Additional file 9: Figure S9. Immune landscape in an unsupervised hierarchical clustering view in GEO cohort (Figure 8) and TCGA cohort (Figure 9). Patients were stratified in four major subtypes: S1 (immune cell inflamed), S2 (immune cell escaped), S3 (immune desert) and a new subtype: S4 (macrophage/monocyte infiltrated). The former three subtypes have been reported by several studies. The mechanism of macrophage/monocyte infiltration in HCC as a unique subtype requires to be further investigated. [file 12935_2021_2108_MOESM9_ESM.pdf]

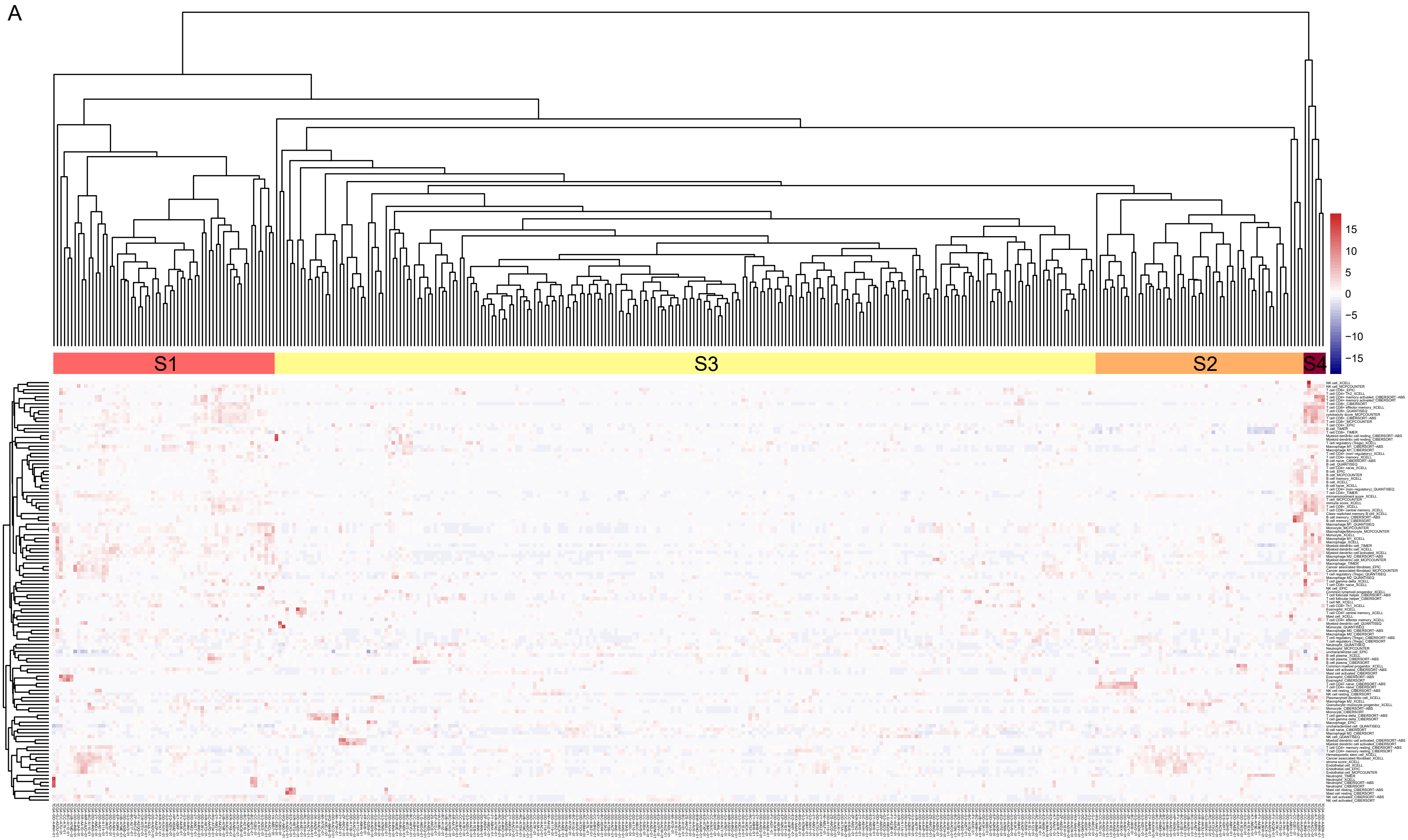

Supplementary Figure 9
