# Supplementary material for: Identification and integrative analysis of ACLY and related gene panels associated with immune microenvironment reveal prognostic significance in hepatocellular carcinoma
Source: Cancer Cell Int. 2021 Aug 3;21:409. doi: 10.1186/s12935-021-02108-2 (PMC8335999; doi:10.1186/s12935-021-02108-2)
Supplement: Supplementary file 11 — Additional file 11: Figure S11. Relationships among ACLY expression level and TIIC level in TCGA cohort. Neutrophils and macrophages seemed to have an increased number of positive correlations in various kinds of malignancies. While infiltration level of NK cell and CD8+ T cell were negatively correlated with ACLY expression. This may explain the reason why high ACLY expression is associated with poor prognosis. Mechanism study is needed for further consolidation. [file 12935_2021_2108_MOESM11_ESM.pdf]

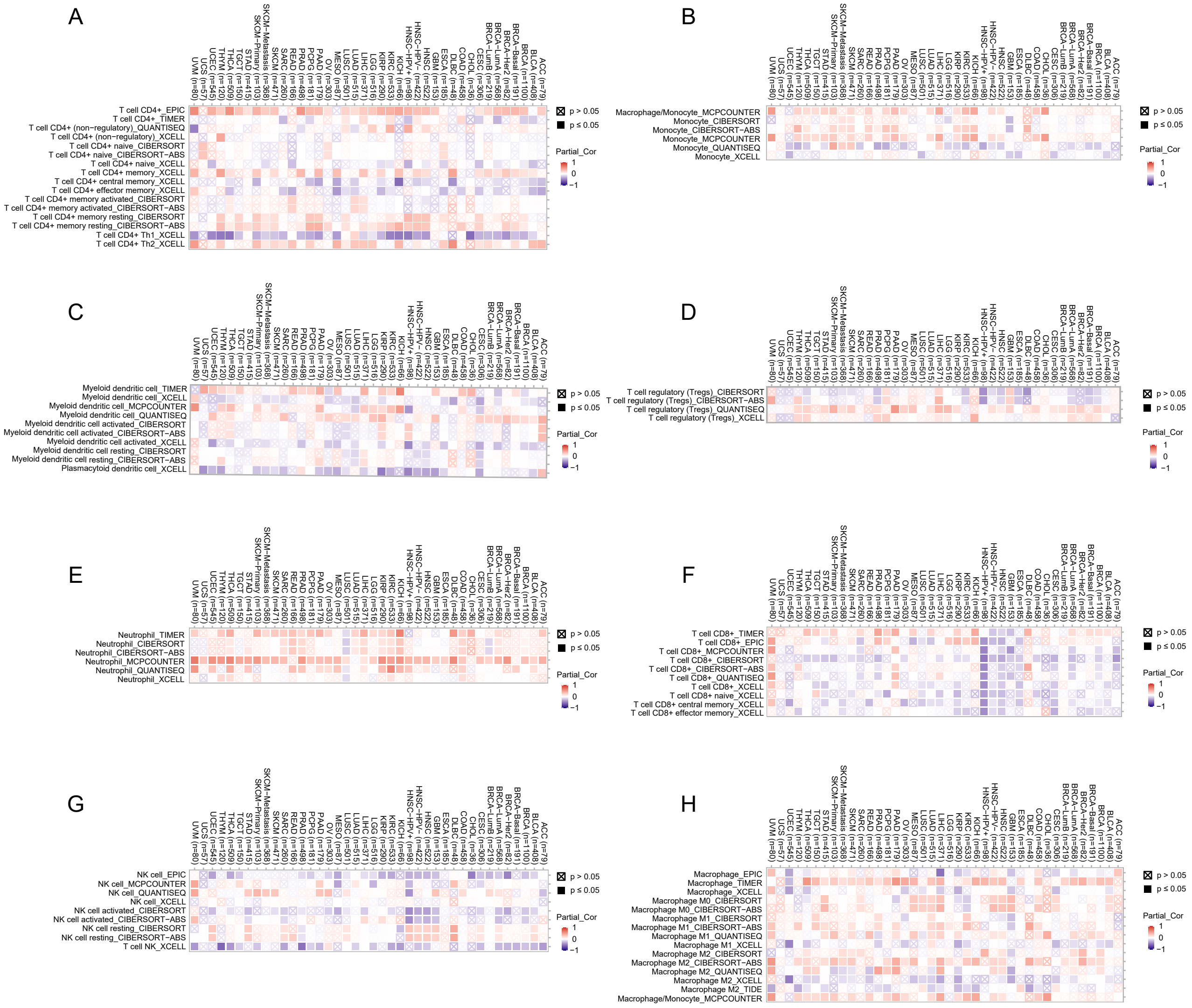

Supplementary Figure 11
